# Intrinsic intermolecular photoinduced charge separation in organic radical semiconductors

**DOI:** 10.1038/s41563-025-02362-z

**Published:** 2025-09-30

**Authors:** Biwen Li, Petri Murto, Rituparno Chowdhury, Laura Brown, Yutong Han, Giacomo Londi, David Beljonne, Hugo Bronstein, Richard H. Friend

**Affiliations:** 1 The Cavendish Laboratory, University of Cambridge, Cambridge, UK; 2 Yusuf Hamied Department of Chemistry, University of Cambridge, Cambridge, UK; 3 Department of Chemistry and Materials Science, Aalto University, Espoo, Finland; 4 Department of Chemical Engineering and Biotechnology, University of Cambridge, Cambridge, UK; 5 Department of Chemistry and Industrial Chemistry, University of Pisa, Pisa, Italy; 6 Laboratory for Chemistry of Novel Materials, University of Mons, Mons, Belgium

**Keywords:** Electronic devices, Electronic and spintronic devices

## Abstract

Organic radicals based on tris(2,4,6-trichlorophenyl)methyl (TTM) radicals show efficient photoluminescence from excitons in the spin-doublet manifold, but their potential in charge photogeneration remains unexplored. Here we report that when TTMs are in contact, photoexcitation generates TTM anion–TTM cation pairs. These can decay radiatively or be fully separated under an electric field bias. We use a triphenyl-substituted TTM (P_3_TTM) in which the phenyl end groups enhance intermolecular interactions. In dilute (5 wt%) films in a wide-energy-gap organic semiconductor host, we observe prompt photoluminescence from the excited radical at 645 nm, and a delayed component, beyond 1 μs, at 800 nm due to recombination of P_3_TTM anion–cation pairs. Measurements of photocurrent made with diode structures with 100% P_3_TTM showed close-to-unity charge collection efficiency in reverse bias. We have found ‘homojunction’ intermolecular charge separation, made possible when the extra energy for double occupancy of the non-bonding radical level on the anion is lower than the energy of the doublet exciton. This opens possibilities for light harvesting using single-material molecular semiconductors.

## Abstract (web summary)

Homojunction intermolecular charge separation is reported in photoexcited anion–cation pairs induced by external electric field bias, opening possibilities for light harvesting using molecular semiconductors.

## Main

Interest in organic radical semiconductors is due to their potential applications in optoelectronic devices, biomedical technologies, quantum information systems and chiral materials^1–10^. Among the organic radicals family, trityl systems are of interest due to their chemical stability and photoluminescence (PL) quantum efficiency^11,12^. These support efficient organic light-emitting diodes (OLEDs), but are generally used as a dilute guest component in a molecular semiconductor ‘host’, since PL is generally quenched at high concentrations when inter-radical interactions are possible. Tris(2,4,6-trichlorophenyl)methyl (TTM) by itself is an alternant hydrocarbon; therefore, the energy degeneracy between the highest occupied molecular orbital (HOMO) to the singly occupied molecular orbital (SOMO) and SOMO to the lowest unoccupied molecular orbital transitions cause weak absorption and slow PL emission rates. Higher efficiencies are achieved with an attached electron donor, including carbazole and triphenylamine^13–15^, where the lowest transition is of intramolecular charge transfer (CT) from donor to TTM. PL quantum efficiencies reported for these systems that emit in the red and near-infrared regimes are often very high, particularly for red and near-infrared emission, and it has recently been shown that multiphonon decay is suppressed for these structures due to reduced vibrational coupling to the exciton^16^. We have also reported a wide range of TTMs with the substitution of *para*-chlorine with phenyl-based-substituents to adjust the colour and efficiency of emission by means of the steric bulk^17–19^. This provides a platform for red and near-infrared OLEDs.

**Fig. 1:**
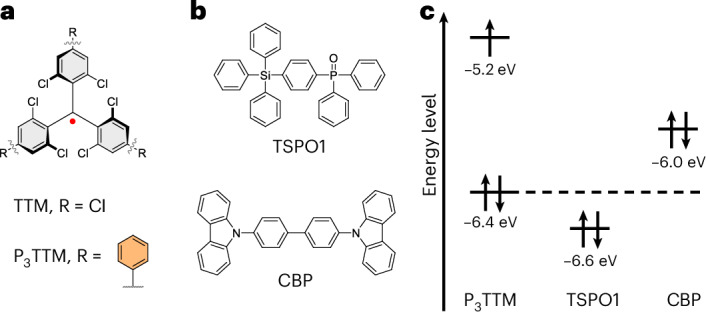
Chemical structures of the investigated molecules and relative molecular orbital energy between radical dopants and host materials. **a**,**b**, Chemical structures of TTM-based radical emitters (**a**) and host materials (**b**) investigated in this study. **c**, Corresponding molecular orbital energy alignment of P_3_TTM and host materials. The host materials only show the HOMO energy (energies are indicative and estimated from reported values^18,24–27^).

**Fig. 2:**
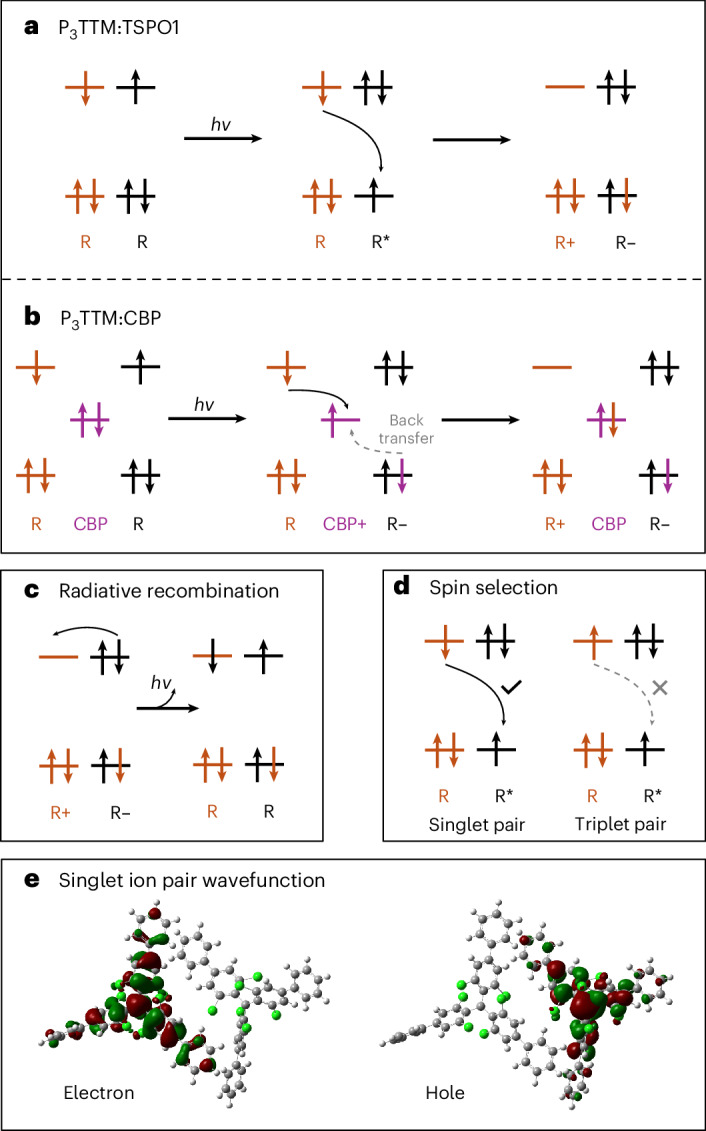
Schematic of CT and P_3_TTM anion and cation recombination process in TSPO1 and CBP. **a**–**d**, Occupancies of the single-particle states; the total ‘exciton’ energy changes that occur when the occupancies of states are changed are not shown. **a**, Intermolecular CT directly takes place between the ground-state radical (R) and excited-state radical (R*) to generate cations (R+) and anions (R–). **b**, The photogenerated hole is quickly transferred to the CBP to form R– first, followed by the second CT process between R and CBP+ that generates R+. **c**, Redshifted emission band is attributed to the electron–hole recombination from the ion pair. **d**, Magnetic field modulates the population of singlet molecular pairs, which is allowed for intermolecular CT, whereas triplet pairs are not able to form R+ and R–. **e**, TDDFT electron–hole wavefunction for a singlet ion pair in the P_3_TTM single crystal. Note how the wavefunctions with major weight on the radical centre extend over the conjugated phenyl groups.

Here we consider the effects of intermolecular interactions between radical and radical (or radical and host). Inter-radical interactions have been reported using the co-crystals of radicals and their hydrogenated radical precursors, allowing good control of inter-radical contacts. A strongly redshifted PL band at room temperature is observed above 5% radical loading, and this shows strong magnetoluminescence below 20 K (ref. ^20^). This is attributed to an excimer, but as noted in a recent review, the propeller shape of the TTM radical should not allow the strong π–π interactions generally required for strong excimer formation with redshifted PL, and that further work is needed to explain this redshifted emission^21^. We present evidence here that redshifted PL may arise from a fully charge-separated anion–cation pair, rather than from a conventional excimer. The materials explored here are shown in Fig. 1. For a regular closed-shell semiconductor, electron–hole generation requires photoexcitation across the semiconductor bandgap. By contrast, as we develop here, for radical semiconductors, electron–hole generation between neighbouring triphenyl-substituted TTMs (P_3_TTMs) can occur just within the SOMO non-bonding orbitals (Fig. 2a), leading to spinless anions and cations. In the absence of onsite Coulomb repulsion energies, this transfer would be barrierless. However, there is an energy cost to do this; it is the charging energy to doubly occupy the anion SOMO. This onsite Coulomb energy is the Hubbard *U* and this is easily measured from the voltage difference for electrochemical oxidation, where the electron is removed from the SOMO to form a cation, versus reduction in which a second electron is added to the SOMO to form an anion. It is large because the SOMO non-bonding orbitals are relatively localized. The reduction and oxidation potentials of P_3_TTM are –1.05 V and + 0.67 V, respectively; therefore, the energy gap is 1.72 eV corresponding to PL at 720 nm, which is close to the broad redshifted emission band^18^.

In this study, we use P_3_TTM (Fig. 1a) in which the phenyl groups are in good conjugation with the TTM core (an average phenyl–phenyl dihedral angle of 34.8° is obtained from the X-ray crystal structure)^18^, giving PL with peak emission at 645 nm, and can provide effective inter-radical contact (Supplementary Section 8). We used a series of hole transport materials widely used in OLEDs as the host matrixes of P_3_TTM. These allow tuning of the host–dopant energy alignment (Fig. 1c) and allow the study of radical–radical and radical–host interactions. We also use solutions in toluene for which different concentrations can be used to control radical–radical interactions.

## Photoinduced charge separation and radiative recombination in P_3_TTM

Radical-doped films were prepared via vacuum sublimation, as used for radical OLEDs^5,13,14,22,23^. In the doping range of 3 wt% to 8 wt%, differences in the observed PL are small and most results presented here are for 5 wt%. We denote films as P_3_TTM:host for a range of hosts used (Fig. 1b), along with an overview of the relative molecular orbital energy alignment of dopant and host materials, based on previously reported values^18,24–27^. We note that these hosts allow tuning of the HOMO energy gap between the dopant and hosts.

**Fig. 3:**
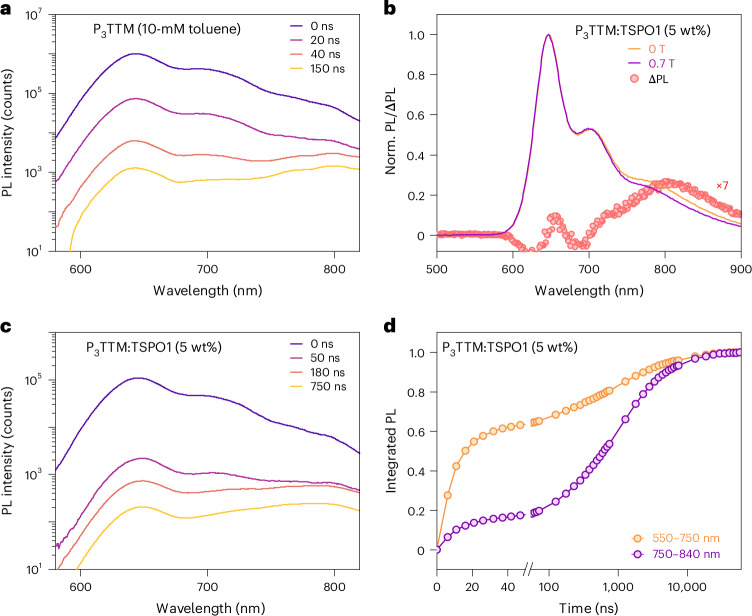
Transient PL and magneto-PL of P_3_TTM in solution and TSPO1. **a**,**c**, Time-resolved PL spectra of concentrated P_3_TTM toluene solution (10 mM; **a**) and P_3_TTM:TSPO1 (5 wt%; **c**). **b**, PL spectra of P_3_TTM:TSPO1 (5 wt%) under fields of 0 T and 0.7 T at room temperature and PL change under magnetic field with respect to the emission wavelength, normalized to the peak emission at 645 nm. **d**, Time evolution of the integrated PL fraction (obtained from the time of the PL spectra) of P_3_TTM:TSPO1 (5 wt%) for molecular emission and redshifted emission band. Source data

Time-resolved PL data with 400-nm excitation for P_3_TTM in dilute and concentrated toluene solutions and TSPO1 films are shown Fig. 3 and Supplementary Fig. 3. In the dilute solution (0.1 mM), the PL peak at 645 nm shows no spectral evolution and a mono-exponential lifetime of 9.1 ns. By contrast, the concentrated 10-mM toluene solution shows a redshifted emission band that becomes dominant at late time (around >40 ns; Fig. 3a). At this concentration, the rate of P_3_TTM–P_3_TTM collisions is high enough to ensure collision within the 9.1-ns exciton lifetime^28^. The associated reduction in the 645-nm PL lifetime with increased concentration follows the Stern–Volmer kinetics, indicating that this is a bimolecular process (Supplementary Section 9). Figure 3c shows the PL results for P_3_TTM:TSPO1. TSPO1 with its wide bandgap provides an inert environment for the radical dopants; therefore, there is no host–dopant interface effect. At early times, only the molecular emission peak at 645 nm from the molecular P_3_TTM exciton is observed. An additional broad emission band beyond 750 nm appears at later times. Figure 3d shows the integrated PL fraction of P_3_TTM:TSPO1. We see that the PL has two main contributions. At 550–750 nm, PL is mainly from the molecular exciton with a lifetime of 11.5 ns, similar to the dilute solution. However, the redshifted emission band at 750–840 nm is long-lived; around 50% of the integrated PL is detected after 500 ns. In contrast to the 645-nm emission band, which changes little ( < 2%), the redshifted emission band shows a strong magnetic field effect (MFE; Fig. 3b). The PL is suppressed at 0.7 T at room temperature and the spectrum for the change in PL intensity, ∆PL = PL (0 T) – PL (0.7 T), shows a broad band extending from 700 nm to a peak at 800 nm. We note that this MFE has been reported for ‘excimer-like’ emissions of other TTM or (3,5-dichloro-4-pyridyl)bis(2,4,6-trichlorophenyl)methyl (PyBTM) based mono- or diradicals, and low-temperature (<20 K) magneto-PL is reported on PyBTM co-crystals^20,29^.

We instead associate the redshifted emission with an inter-P_3_TTM CT recombination (Fig. 2c). First evidence for this comes from quantum-chemical calculations performed on close molecular pairs of the P_3_TTM single crystal (Supplementary Section 7). Time-dependent density functional theory (TDDFT) calculations indeed show the presence of intermolecular singlet CT states lying ~0.4 eV below the localized P_3_TTM excitons, in excellent agreement with the energy difference (~0.3–0.5 eV) inferred from the two main measured PL features (Fig. 3). As we hypothesized, there is substantial extension of SOMOs onto the peripheral phenyl rings (Fig. 2e) that provides the needed electronic couplings for the generation of CT pairs from the optically excited excitons and the (non-)radiative recombination of these CTs to the ground state. Applying Marcus-like rate theory to the results of TDDFT calculations performed for close pairs in the crystal yields charge generation rates up to 1 ps^−1^ and CT lifetimes approaching the microsecond range (Supplementary Section 7). Thus, our calculations suggest the fast formation of long-lived CT pairs that, in the absence of competing decay mechanisms, should enable efficient free charge generation, as confirmed below.

**Fig. 4:**
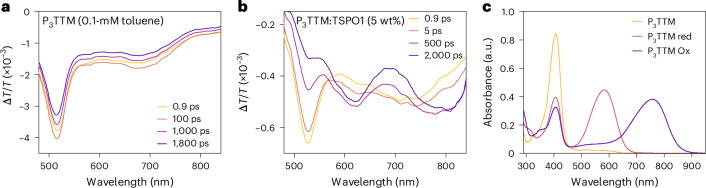
TA measurements and spectroelectrochemistry of P_3_TTM in solution and TSPO1. **a**,**b**, Picosecond-scale visible TA spectra of diluted P_3_TTM toluene solution (0.1 mM; **a**) and P_3_TTM:TSPO1 (5 wt%; **b**). *λ*_ex_ = 400 nm, 17–27 μJ cm^−2^ per pulse. **c**, Spectroelectrochemistry of P_3_TTM in a degassed tetrahydrofuran solution, showing P_3_TTM, reduced P_3_TTM (P_3_TTM red) and oxidized P_3_TTM (P_3_TTM Ox). Source data

Transient optical absorption (TA) measurements are shown in Fig. 4. For a dilute toluene solution in the visible probe region (Fig. 4a), a broad photoinduced absorption (PIA) signal peaked at 675 nm and a sharp PIA in the region of 480–550 nm corresponding to the radical D_1_ state is probed^5,12^. There is no spectral shape change observed in the late time, matching the result of the time-resolved PL. However, for P_3_TTM:TSPO1 (Fig. 4b), two distinct PIA peaks (570–630 nm and 730–850 nm) grow, and the PIA of the radical D_1_ state reduces with time. We have carried out steady-state spectroelectrochemistry measurements (Fig. 4c). We observe absorptions of anions at 580 nm and cations at 760 nm and a bleaching of the ground-state absorption at 407 nm for the D_0_–D_2_ transition^13^. These features from spectroelectrochemistry map well with the TA features, and we associate these PIA states to closed-shell anions (570–630 nm) and cations (730–850 nm). The concentrated P_3_TTM solution (10 mM) shows similar long-time TA (Supplementary Figs. 9 and 10), although the CT kinetics are slower in the solution since molecular diffusion and collision need to be considered^28^. We note that CT can only proceed for overall spin singlet excited P_3_TTM (P_3_TTM*)–P_3_TTM pairs (Fig. 2d). This is consistent with the strong magneto-PL shown in Fig. 3b, and we consider that the applied magnetic field modulates the population of triplet and singlet sublevels. The reduction in singlet population with the applied field is consistent with the level crossing at the exchange energy, noting that we expect an antiferromagnetic ground state. Larger values of magnetoluminescence are reported at low temperatures and higher magnetic fields^20^. We emphasize that the CT between P_3_TTM radical pairs is very different to the standard electron–hole transfer in closed-shell semiconductors. As shown in Fig. 2a, both electrons and holes are in the SOMO levels of the two radicals, rather than the HOMO and lowest unoccupied molecular orbital with an energy gap. We note that this intermolecular CT exciton can be seen as an intermolecular analogue of the zwitterionic excited state of di- or polyradicals^30,31^.

**Fig. 5:**
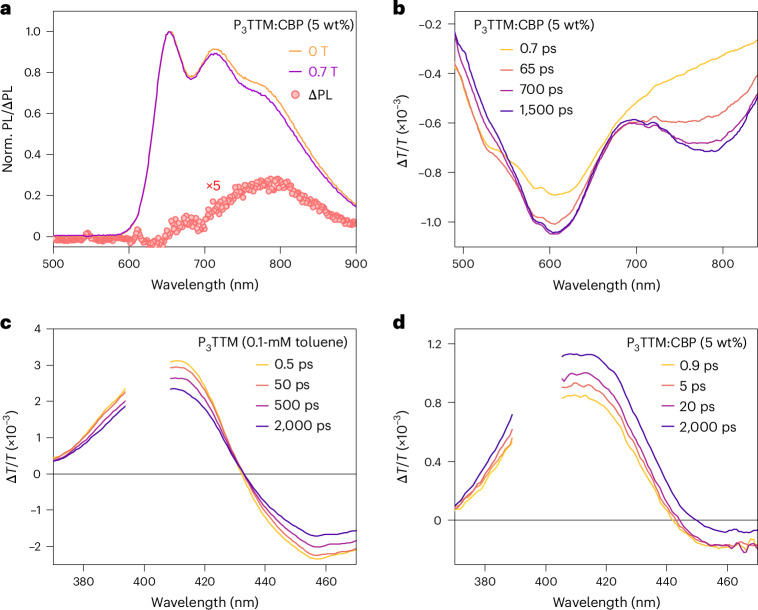
Magneto-PL and transient spectroscopies of P_3_TTM in CBP. **a**, PL spectra of P_3_TTM:CBP (5 wt%) under fields of 0 T and 0.7 T at room temperature and PL change under magnetic field with respect to the emission wavelength, normalized to the emission at 645 nm. **b**, Visible TA spectrum of P_3_TTM:CBP. **c**,**d**, Ultraviolet TA spectra of diluted P_3_TTM toluene solution (0.1 mM; **c**) and P_3_TTM:CBP (5 wt%; **d**). The spectrum break is due to pump laser scattering (*λ*_ex_ *=* 400 nm, 12–13 μJ cm^−2^ per pulse). Source data

We have previously reported a host–dopant intermolecular CT state between mesitylated TTM, M_3_TTM and a donor CBP^32^ host (structure shown in Fig. 1), which gives long-lived emission, where the TTM acts as an electron acceptor^17^. Figure 5 shows the effects of CBP host matrixes on intermolecular CT. The PL of a P_3_TTM:CBP system is shown in Fig. 5a, exhibiting similar behaviour as the P_3_TTM:TSPO1 system, with a magnetic-field-dependent redshifted band centred near 790 nm, which is also time delayed (Supplementary Fig. 5). This indicates broadly similar behaviour as the P_3_TTM:TSPO1 system, but as we explore below, there is an intermediate time regime in which CBP has transferred an electron to the P_3_TTM. This can then be followed by either back transfer from the P_3_TTM anion (causing a longer molecular emission lifetime of 13.7 ns; Supplementary Fig. 8) and subsequent electron transfer to CBP from a neutral P_3_TTM.

The visible TA spectrum shown in Fig. 5b of the CBP film has two similar broad PIA peaks in the same region of 570–630 nm and 730–850 nm as the TSPO1 film, as the same radical anions and cations are generated, but the 730–850-nm band (associated with the P_3_TTM cation) grows in more slowly over 100 ps. Given the 5% P_3_TTM fraction, the CT process most accessible will be from CBP to P_3_TTM, and we attribute the slower build-up of the P_3_TTM cation population to the subsequent CT to bring CBP back to zero charge. Access to the CBP–P_3_TTM CT process allows a faster initial growth of the P_3_TTM anion population compared with the P_3_TTM:TSPO1 system. We also note that the first CT process between CBP and P_3_TTM* is not spin selective; spinless CBP–P_3_TTM* pairs give quicker CT kinetics, but the subsequent CT between positively charged CBP, CBP^+^ and P_3_TTM is dependent on the spin state of the CBP^+^–P_3_TTM pairs. Therefore, the PL of P_3_TTM:CBP also has an MFE for the redshifted emission band (Fig. 5a). In both TSPO1 and CBP, the growth rate of the anion and cation PIA increases for a higher doping concentration or closer contact between radicals. The CT population build-up times are shortened to several picoseconds in the 100-wt% doped film (without hosts) due to a short inter-radical distance (Supplementary Figs. 30 and 31).

TA of the ground-state bleach (GSB) for P_3_TTM (360–430 nm) is shown for dilute solutions in Fig. 5c and for P_3_TTM:CBP films in Fig. 5d. For the dilute solution, GSB is the largest at early times and its later decay is consistent with the 9.1-ns PL decay. By contrast, the P_3_TTM:CBP film shows GSB growth up to 2 ns. The early time GSB is due to the P_3_TTM exciton evolving into P_3_TTM anions, accompanied by CBP cations. The GSB growth is due to the conversion of CBP cations to P_3_TTM cations as the full P_3_TTM anion–cation population develops. In the first 2 ns, although the GSB for the dilute solution falls by 27%, for the P_3_TTM:CBP film, the GSB rises by 32%. This indicates that the quantum yield for charge photogeneration is remarkably high, which we estimate to be up to 40% (Supplementary Section 5).

## Direct-charge photogeneration

Our studies of P_3_TTM in solution and in solid hosts indicate clear evidence for a fully charge-separated anion–cation intermolecular state, which shows PL near 800 nm. Excitation fluence measurements (Supplementary Fig. 7) indicate that this anion–cation pair is mostly bound at room temperature. We have investigated solid films of P_3_TTM without a host material in standard diode device structures, showing that it is possible to fully separate electrons and holes with an applied field. The films show substantially reduced PL (Supplementary Table 4) and tracked through reduced photoexcited-state lifetimes (Supplementary Figs. 30 and 31). The TA spectra shown in Supplementary Fig. 32 reveal clear evidence for early time charge separation, with anion (600 nm) and cation (800 nm) bands fully formed by 4 ps, but rapid decay to the ground state within a few nanoseconds. In spite of these reduced lifetimes, we are still able to get long-range charge separation in biased diode structures.

**Fig. 6:**
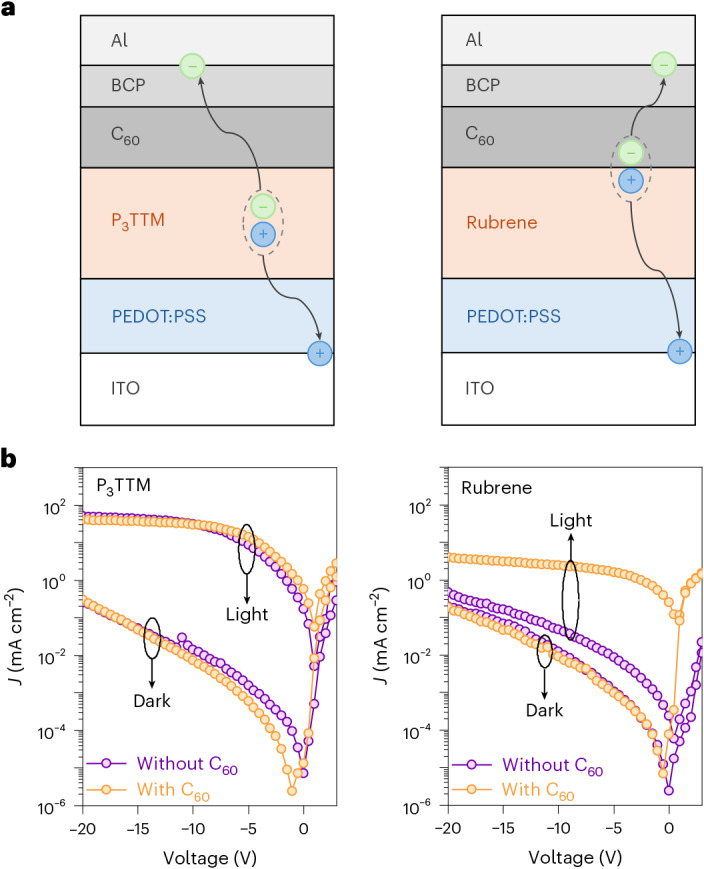
Photocurrent measurements of P_3_TTM and rubrene-based devices. **a**, Device architecture and schematic of the charge separation process in the P_3_TTM device (left) and rubrene device (right). **b**, Photocurrent density under 395-nm excitation at 160 mW cm^−2^ and dark current density (*J*) comparison of the P_3_TTM device (left) and rubrene device (right). Source data

We fabricated standard multilayer diode structures using poly(3,4-ethylene dioxythiophene):poly(styrene sulfonate) (PEDOT:PSS) on indium tin oxide (ITO) as the hole injection/extraction electrode, and fullerene (C_60_)/bathocuproine (BCP)/aluminium (Al) as the electron injection/extraction layers. These were selected to give band alignment with the P_3_TTM radical. We also made devices using rubrene as a control, since this has similar redox potentials. The device architecture used is shown in Fig. 6a for ITO (150 nm)/PEDOT:PSS (40 nm)/photoactive layer (P_3_TTM or rubrene) (80 nm)/C_60_ (20 nm)/BCP (5 nm)/Al (100 nm). Figure 6b shows the photocurrent and dark current under bias for both P_3_TTM and rubrene devices (both made with and without the C_60_ layer and photoexcitation at 395 nm was 160 mW cm^−2^).

The rubrene device shows expected behaviour for charge photogeneration at the rubrene/C_60_ heterojunction, giving a short-circuit current density of 0.25 mA cm^−2^ and a quantum yield of 4.9%, considering 90% transmission rate of the rubrene thin film under ultraviolet (UV) light. This is consistent with charge photogeneration within an exciton diffusion range of 6–8 nm in rubrene^33^. Under reverse bias, these devices showed little current increase, rising to 4 mA cm^−2^ at –20 V. For diodes made without the C_60_ layer, the photocurrents are much lower, similar in magnitude to the dark current, showing that the rubrene/BCP heterojunction does not generate free charge carriers.

By contrast, the P_3_TTM diode showed a low quantum yield at short circuit, but a very strong increase in reverse bias that saturates at around 45 mA cm^−2^, indicating a quantum yield for charge collection close to 100% and considering a 15% transmission rate of the P_3_TTM thin film under UV light. The behaviour was very similar for devices made with and without the C_60_ layer, indicating that charge photogeneration was not controlled by the P_3_TTM/C_60_ interface. Rather, this close-to-unity charge collection efficiency is due to bulk charge photogeneration, as described above.

The ability to fully separate electrons and holes following photoexcitation in a diode structure made with a single non-polar semiconductor is routine in mainstream inorganic semiconductors such as silicon, in which the photogenerated exciton is large and has low binding energy (<10 meV), but for molecular systems, this has rarely been previously reported. We note that there are interesting studies on ‘symmetry-breaking’ CT at an intramolecular level, often in the presence of a polar solvent^34–39^. We also note that our demonstrated high-efficiency of charge photogeneration precludes any ‘excimer’ formation, which typically do not undergo charge separation.

## Outlook

In summary, the intermolecular interaction and excited-state dynamics of neutral π radicals are studied by various time-resolved techniques and steady-state spectroscopies. In both solutions and films, we identified an intermolecular symmetry-breaking charge separation process between P_3_TTM SOMOs to form an excited-ion pair state comprising a pair of closed-shell anion and cation. The GSB growth of P_3_TTM provides further evidence for the intermolecular process. We also demonstrated that the redshifted PL has an MFE, as the intermolecular CT depends on the spin state of P_3_TTM*–P_3_TTM and CBP^+^–P_3_TTM intermediates. This mechanism can be generalized to other open-shell materials, for example, the long-lived and redshifted emission generally observed for TTM- and PyBTM-based radicals can be attributed to the recombination of this CT exciton. We also explored the effect of host–dopant interface on the intermolecular CT channels. This work demonstrates that the photoinduced charge separation can be intermolecularly driven in neutral radicals and that the unpaired electron can be mobile and not bound to the radical centre. Homojunction charge separation, as observed here, has been a long-sought-after goal in organic photovoltaics^40^. This work provides an avenue for the exploration of power generation and solar-driven chemistry in both solution and solid state using only a single component.

## Methods

### Film preparation

Radicals were synthesized as previously reported^18^. Host materials were obtained from Ossila without further purification. Thin films were prepared by thermal evaporation under vacuum (~10^−7^ torr, Angstrom Engineering EvoVac 700 system). Here 100 nm of 5 wt% radical-doped CBP, TSPO1 and TAPC films were deposited on UV fused silica substrates. Polymethyl methacrylate films were prepared by spin coating in a nitrogen-filled glovebox. All the films were encapsulated in this glovebox. The doping concentration stated in this study denotes the weight percentage.

### Device fabrication and characterization

Organic transport layer materials were obtained from Ossila without further purification. The ITO substrate was cleaned in an ultrasonic bath of detergent, deionized water, acetone and isopropanol for 10 min each. It was then UV treated in a UV–ozone chamber for 12 min. A thin layer of PEDOT:PSS (Clevios P VP AI 4083) was prepared by spin coating the PEDOT:PSS solution at 3,000 rpm for 40 s on the ITO substrate and annealed at 150 °C for 15 min in air. Layers of P_3_TTM, C_60_, BCP and Al were deposited by thermal evaporation under a vacuum (~10^−7^ torr, Angstrom Engineering EvoVac 700 system) at a rate of 0.1–1 Å s^−1^. The whole device has a structure of ITO (150 nm)/PEDOT:PSS (40 nm)/P_3_TTM (80 nm)/C_60_ (20 nm)/BCP (5 nm)/Al (100 nm). A Keithley 2635A source with a 395-nm light source was used to measure the current–voltage plot. The voltage was applied to the device between −20 V and 3 V at a sweep speed of 0.5 V s^−1^. The effective electrode overlap area was 4.5 mm^2^, which was used to calculate the current density.

### Steady-state photophysics measurements

The absorption spectra were measured by a commercially available Shimadzu UV-2550 spectrophotometer and a Shimadzu UV-1800 spectrophotometer. PL was measured by a custom-made setup by providing continuous photoexcitation at 405 nm from a laser diode. The PL from the samples was collected in a collimating two-lens apparatus and directed into an optical fibre that supplies photons into a calibrated grating spectrometer (Andor SR-303i) and finally into a silicon camera where it is recorded.

### Transient PL spectroscopy

Transient PL (time-resolved PL) spectra at nanosecond–microsecond timescales were recorded using an electronically gated intensified charge-coupled device camera (Andor iStar DH740 CCI-010) connected to a calibrated grating spectrometer (Andor SR-303i). A narrowband non-colinear optical parametric amplifier pumped with a frequency-doubled output of a 1-kHz 800-nm laser pulse (100-fs duration) from a Ti:sapphire amplifier (Spectra Physics Solstice Ace) was used to generate a tuneable excitation pulse. A 400-nm excitation can be achieved by the second harmonic of the 800-nm output, generated using a β-barium borate crystal. Here 425-nm long-pass filters (Edmund Optics) were used to prevent scattered laser signals from entering the spectrometer. Temporal evolution of the emission was obtained by stepping the intensified charge-coupled device delay with respect to the excitation pulse, with a minimum gate width of 5 ns. Recorded data were corrected to account for filter transmission and camera sensitivity.

### TA spectroscopy

The picosecond-scale TA spectroscopies were carried out either on a custom-made TA setup or a commercialized TA setup. The custom-made TA setup has an output from a Ti:sapphire amplifier (Spectra Physics Solstice Ace) that generated 100-fs-duration pulses centred at 800 nm with a 1-kHz repetition rate. The pulse was split into pump and probe beams. The 400-nm excitation pump was generated by passing the second harmonic of the 800-nm output using a β-barium borate crystal. The pump light was chopped at 500 Hz by a chopper wheel. The visible probe light was generated via non-collinear optical parametric amplifiers. The pump–probe time delay was provided by a mechanical delay stage (Thorlabs DDS300-E/M). The probe pulses were split into two beams by a 50/50 beamsplitter to provide a reference beam, which increases the signal-to-noise ratio. The probe pulses were detected by a silicon (Hamamatsu S8381-1024Q) dual-line array with a custom-built board from Stresing Entwicklungsbüro. The fundamental laser beam of the commercialized picosecond-scale TA setup at 1,030 nm was provided by PHAROS. The ORPHEUS system, which was pumped by an ytterbium-doped solid-state chirped pulse amplifier, generated pump lights using 90% of the fundamental laser (LIGHT CONVERSION). The pump wavelength can be varied from 350 nm to 2,000 nm with a 10-kHz repetition rate and an ~100-fs pulse duration. The rest of the 10% fundamental beam was injected into HARPIA-TA to generate the probe light. The pump–probe time delay was provided by a mechanical delay stage. The probe pulses were detected by a spectrograph with dual outputs to cover the UV–near-infrared regions (Kymera-193i-B2).

### Magneto-PL measurement

The encapsulated film sample was positioned between magnet cores (GMW 3470 electromagnet). Its PL spectrum was recorded with an Andor SR-303i spectrometer with and without a magnetic field. The magnetic field was swept from 0 T to 0.7 T with a ramping step of 0.01 T at room temperature. The continuous photoexcitation at 405 nm was provided by a laser diode.

### Spectroelectrochemistry

A commercially available PalmSens EmStat4S potentiostat was connected to a commercially available Shimadzu UV-1800 spectrophotometer. The measurements were carried out in a custom-made three-electrode setup using a quartz cuvette (0.1-mm path length) as the spectroelectrochemical cell, a coil of platinum wire as the working electrode (in the light path), a platinum wire as the counter electrode and a freshly activated silver wire as the Ag/Ag^+^ reference electrode. The silver wire was activated by immersing in a concentrated HCl solution to remove any silver oxides or other impurities, then rinsed with water and acetone and dried before measurements. The reference electrode was calibrated against ferrocene/ferrocenium (Fc/Fc^+^) redox couple. For this setup, Fc/Fc^+^ half-wave potential (*E*_1/2_) was determined at 0.40 V versus Ag/Ag^+^. The supporting electrolyte was 0.1-M solution of Bu_4_NPF_6_ in anhydrous tetrahydrofuran. The electrolyte was bubbled with Ar gas before measurements to remove any dissolved oxygen and the measurements were carried out under an Ar atmosphere. The sample concentration was adjusted to keep the optical density below 1.0 a.u. for neutral radicals. The absorption spectra of the oxidized (cationic) and reduced (anionic) species were measured by applying a constant potential of +1.1 V and –1.4 V versus Fc and Fc^+^, respectively. These are ~0.4 V higher than the corresponding half-wave potentials determined for the P_3_TTM radical^18^.

### Quantum-chemical calculations

Molecular pairs in close contact selected from the X-ray crystal structure of P_3_TTM were used for the calculation of the excited-state properties (vertical excitations, transition dipole moments and state dipoles) by means of TDDFT within the Tamm–Dancoff approximation^41^, in conjunction with the LC-*ωh*PBE functional and the 6-311G(d,p) basis set^42^. Furthermore, to implicitly take into account dielectric screening effects, the screened range-separated hybrid procedure was applied^43^. In such a scheme, the range-separation parameter *ω* was set to 0.100 Bohr^−1^, whereas the other parameters (*α* and *β*) were modified according to the dielectric constant of the solvent/environment. In this work, we chose a dielectric constant typical of toluene, that is, *ε* = 2.37. Regarding the electronic coupling calculations and the (non-)radiative charge dissociation/recombination rates (Supplementary Information), all the calculations were performed using the Gaussian 16 package.

## Online content

Any methods, additional references, Nature Portfolio reporting summaries, source data, extended data, supplementary information, acknowledgements, peer review information; details of author contributions and competing interests; and statements of data and code availability are available at 10.1038/s41563-025-02362-z.

## Supplementary information

Supplementary Information Supplementary Sections 1–11, Figs. 1–35, Tables 1–4 and discussion.

## Source data

Source Data Fig. 3 Source data for Fig. 3.

Source Data Fig. 4 Source data for Fig. 4.

Source Data Fig. 5 Source data for Fig. 5.

Source Data Fig. 6 Source data for Fig. 6.

## Data Availability

The optical spectroscopy and device characterization data generated in this study have been deposited in the University of Cambridge Repository at 10.17863/CAM.120958. Source data are provided with this paper.
