# Supplementary material for: Intrinsic intermolecular photoinduced charge separation in organic radical semiconductors
Source: Nat Mater. 2025 Sep 30;25(1):100–6. doi: 10.1038/s41563-025-02362-z (PMC12747885; doi:10.1038/s41563-025-02362-z)
Supplement: Supplementary file 1 — Supplementary Sections 1–11, Figs. 1–35, Tables 1–4 and discussion. [file 41563_2025_2362_MOESM1_ESM.pdf]

nature materials

Supplementary information https://doi.org/10.1038/s41563-025-02362-z

Intrinsic intermolecular photoinduced charge separation in organic radical semiconductors

In the format provided by the authors and unedited

Table of Contents

S1. Steady state absorption and photoluminescence of P_3TTM in solution and film........2

S2. Transient photoluminescence of P_3TTM in solution 3

S3. Transient photoluminescence of P_3TTM in different host materials 4

S4. Transient absorption of P_3TTM in solution 7

S5. Transient absorption of P_3TTM in film 8

S6. Magneto-photoluminescence of P_3TTM..13

S7. Quantum-chemical calculations 14

S8. X-Ray crystallography 22

S9. The concentration effect on the intermolecular charge transfer.................................23

S10. Device characterisation 28

S11. Reference...30

S1. Steady state absorption and photoluminescence of P_3TTM in solution and film

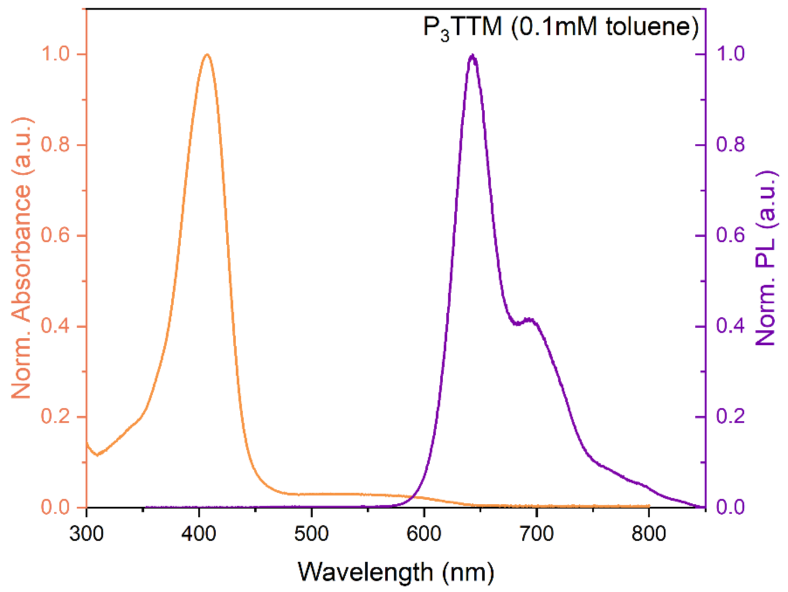

Supplementary Fig. 1 Absorption and steady-state photoluminescence (PL) spectra of P_3TTM in 0.1 mM diluted solution (λ_{ex}= 400 nm). The absorption spectrum has a strong peak at 407 nm of D_0-D_2 transition, and weak absorption signal beyond 480 nm for D_0-D_1 transition. The PL peaked at 645 nm and has a strong vibronic shoulder at 695 nm.

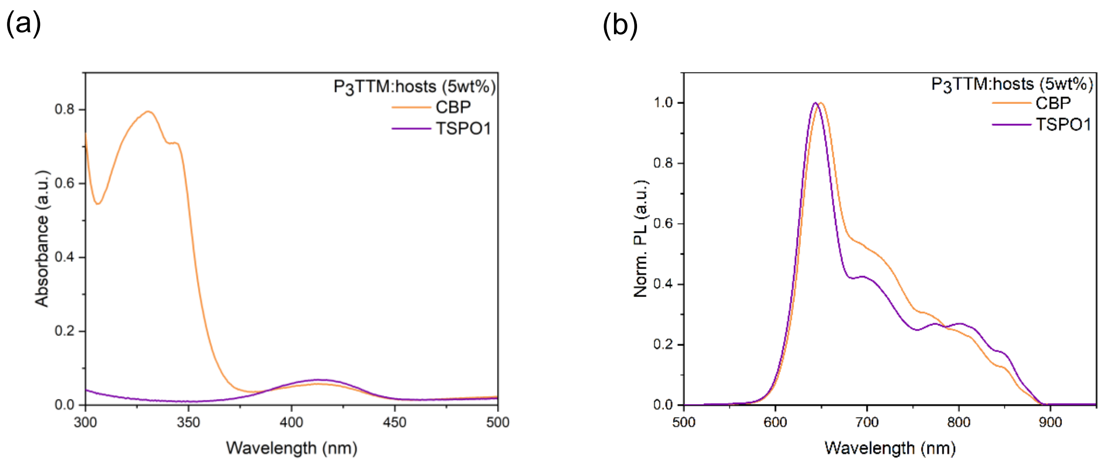

Supplementary Fig. 2 (a) Absorption spectra and **(b)** steady-state PL spectra of P_3TTM:CBP and P_3TTM:TSPO1 (λ_{ex}= 400 nm).

S2. Transient photoluminescence of P_3TTM in solution

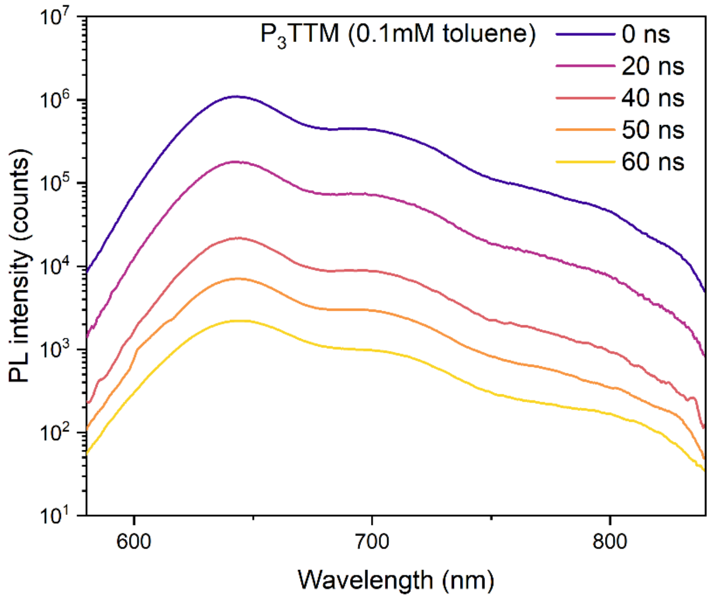

Supplementary Fig. 3 The TrPL spectra of diluted P_3TTM toluene solution (0.1mM), which only shows the molecular doublet emission with a mono-exponential decay (λ_{ex}= 400 nm).

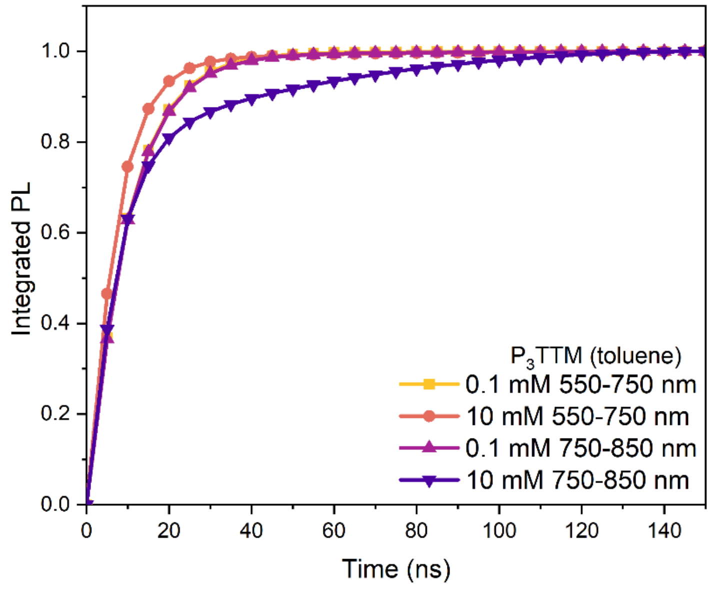

Supplementary Fig. 4 Integrated PL fraction of P_3TTM in toluene solutions with different concentrations (0.1mM *vs.* 10mM). The red-shifted PL band in the 10mM solution has a slower PL kinetics, indicating it originates from a different excited species.

S3. Transient photoluminescence of P$_3$TTM in different host materials

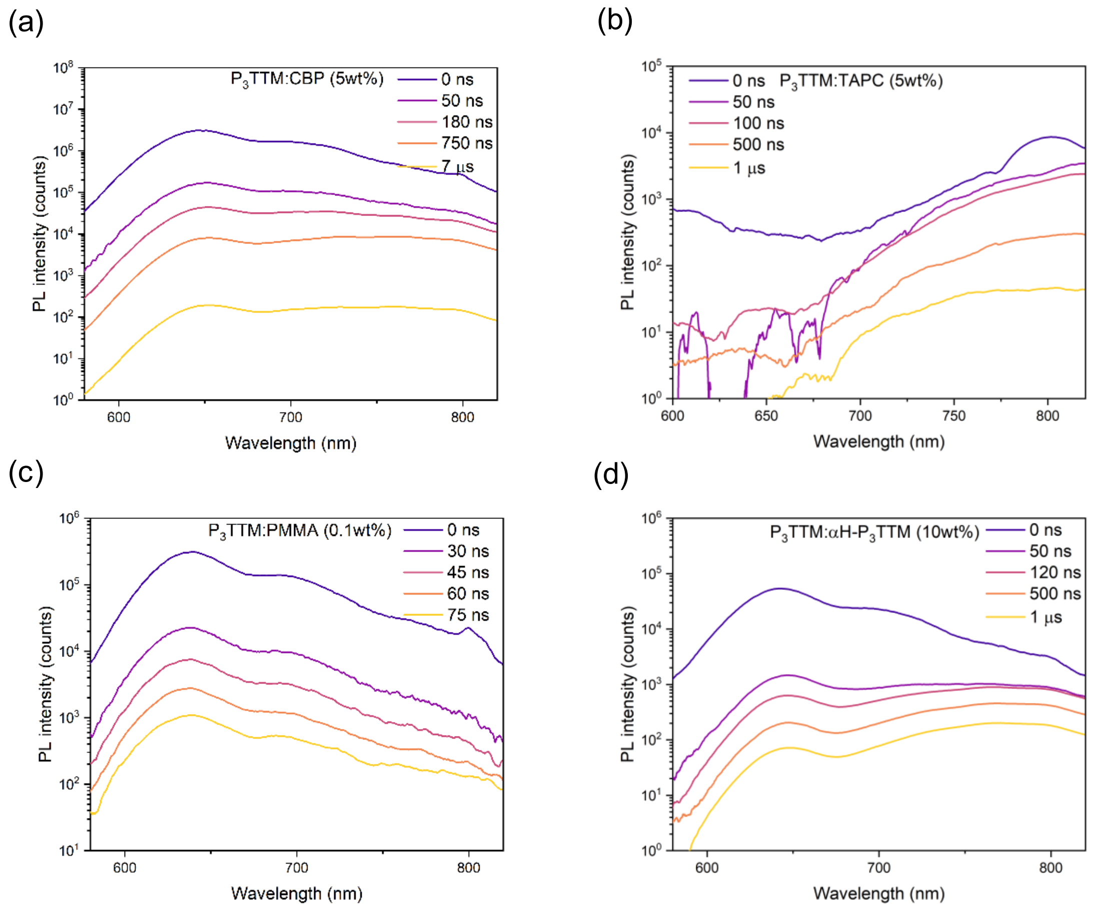

Supplementary Fig. 5 The TrPL spectra (λ_{ex}= 400 nm) of P$_3$TTM doped in **(a)** CBP (5wt%), **(b)** TAPC (5wt%), **(c)** PMMA (0.1 wt%) and **(d)** αH-P$_3$TTM (10 wt%). Similar to the TSPO1 film, the CBP and αH-P$_3$TTM film have a molecular doublet emission peak at 645 nm and an additional red-shifted emission band in the late time. As for the PMMA film with a very low doping concentration, its TrPL is similar to the diluted solution, we only observe a molecular emission with a monoexponential decay kinetic (lifetime at 10.7 ns). However, the molecular emission is completely quenched in TAPC, only exciplex emission beyond 700 nm is probed.

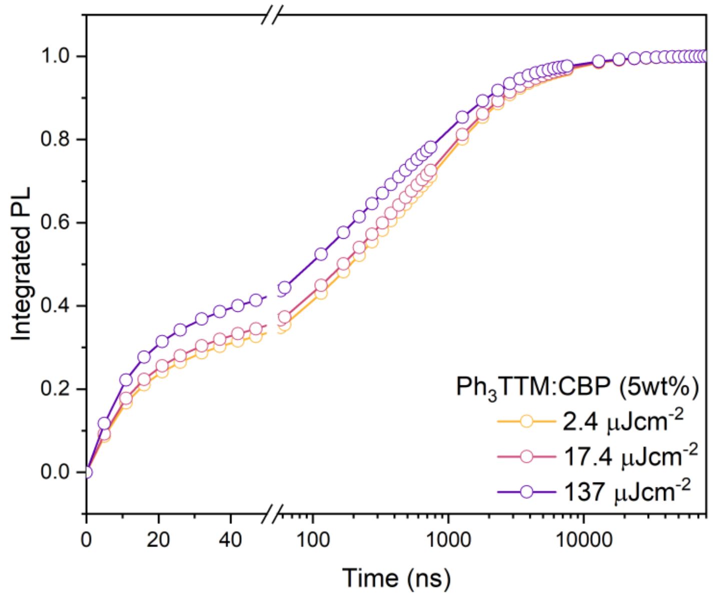

Supplementary Fig. 6 Fluence-dependent TrPL (in the emission region of 550-850 nm) of P3TTM:CBP (λ_{ex}= 400 nm).

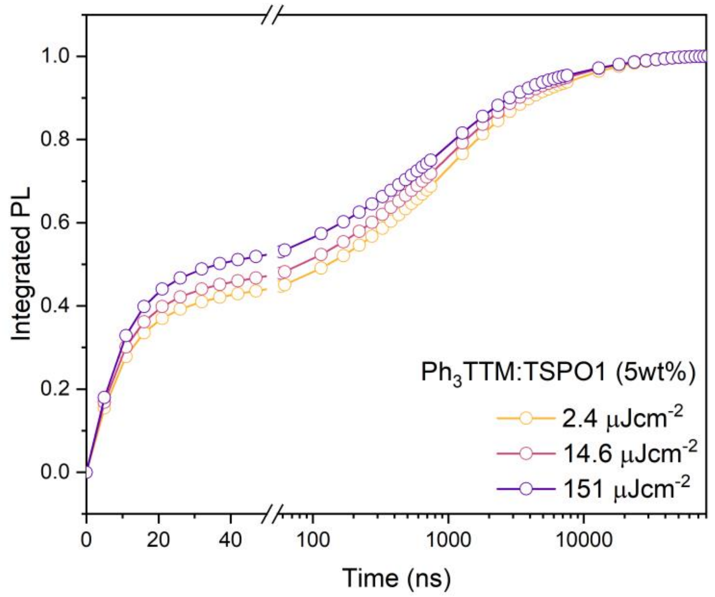

Supplementary Fig. 7 Fluence-dependent TrPL (in the emission region of 550-850 nm) of P3TTM:TSPO1 (λ_{ex}= 400 nm).

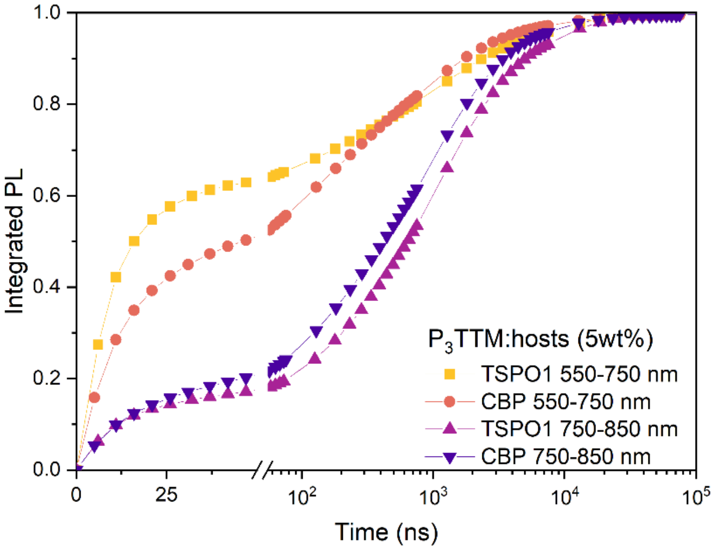

Supplementary Fig. 8 A comparison of integrated PL fraction of P3TTM in TSPO1 and CBP for molecular doublet emission and red-shifted emission. The molecular emission of P3TTM in CBP is slower due to the exciton regeneration process. The red-shifted emission in both films has the same kinetics, implying it is from the same excited species.

Equation used to calculate of integrated PL:

$$integrated\ PL = \frac{\int_0^t PL(t)dt}{\int_0^\infty PL(t)\,dt}$$

S4. Transient absorption of P_3TTM in solution

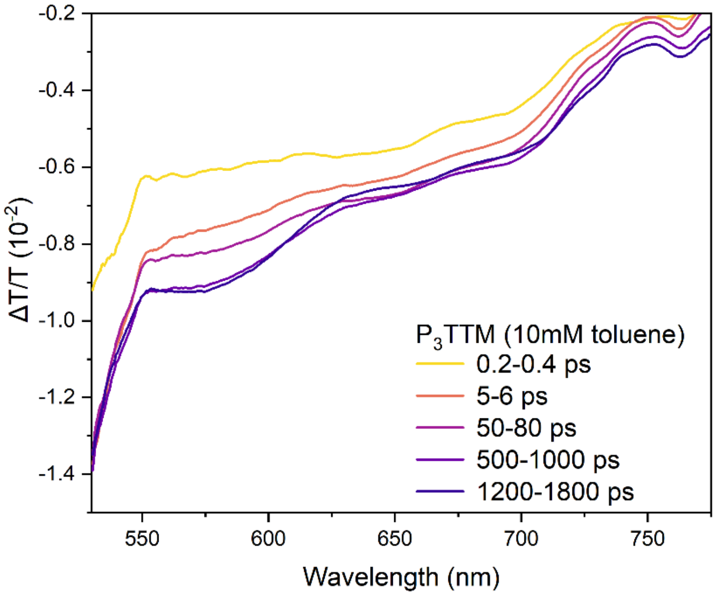

Supplementary Fig. 9 ps TA spectrum (λ_{ex}= 400 nm, 84 µJcm^{-2} per pulse) of P_3TTM (10mM toluene). Molecular D_1 PIAs are probed in the early time, while anions and cations PIA are not clearly seen.

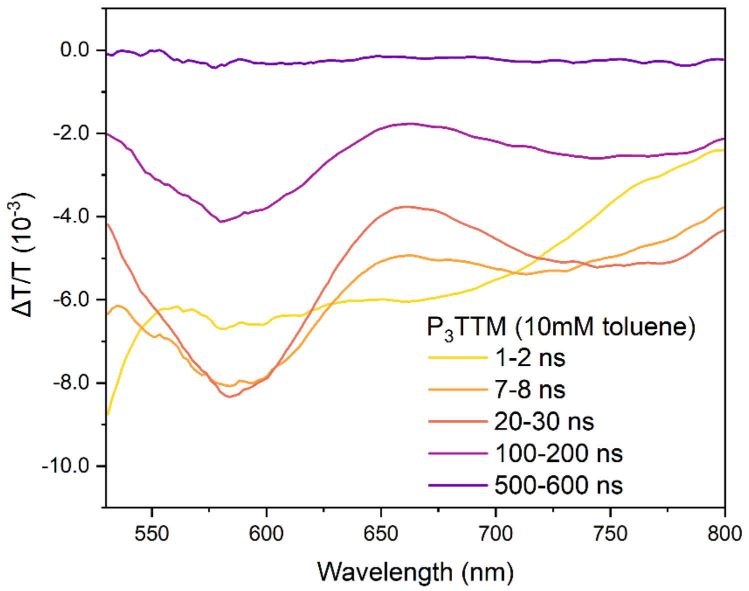

Supplementary Fig. 10 ns TA spectrum (λ_{ex}= 532 nm, 52 µJcm^{-2} per pulse) of P_3TTM (10mM toluene). PIA states of D_1 appear in the early time (1-2 ns), following a PIA of anions (580 nm) and cation (760 nm) generation process within 7-8 ns.

S5. Transient absorption of P_3TTM in film

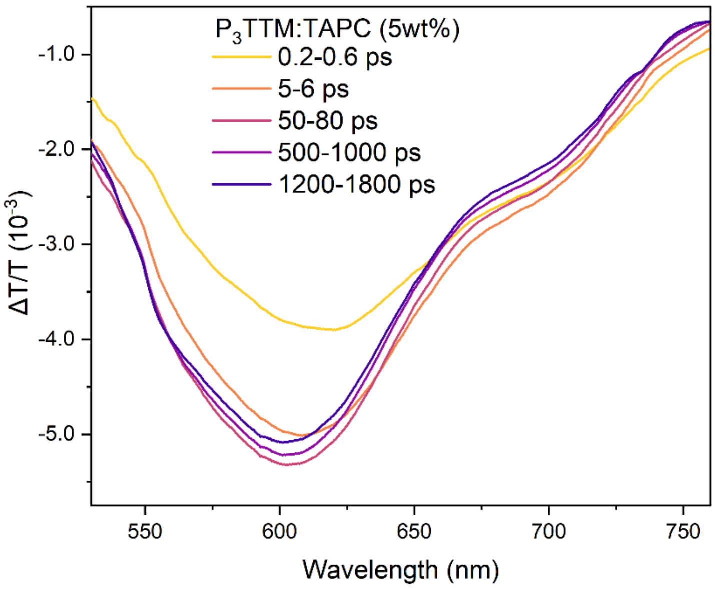

Supplementary Fig. 11 ps TA spectrum in the visible region of P_3TTM:TAPC (5wt%) (λ_{ex}= 400 nm, 84 μJcm^{-2} per pulse). Due to the shallow HOMO of TAPC, photogenerated holes are transferred to host quickly to form host-dopant exciplex. Only anion PIA signal is probed at 610 nm, while molecular D_1 and cations PIA signals are not seen.

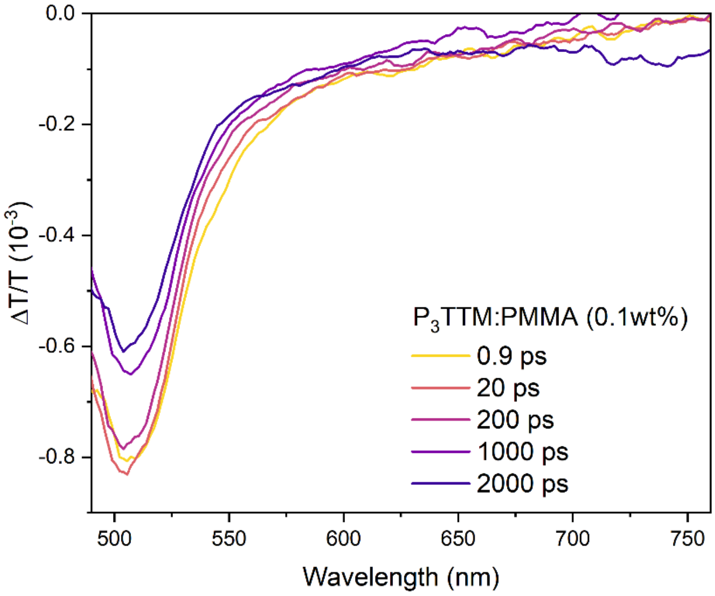

Supplementary Fig. 12 ps TA spectrum in the visible region of P_3TTM:PMMA (0.1wt%) (λ_{ex}= 400 nm, 27 μJcm^{-2} per pulse). Only PIA of molecular D_1 is probed, while radical anions and cations PIAs are not seen in the solid state.

We have also explored other host materials. 1,1-Bis[(di-4-tolylamino)phenyl]cyclohexane (TAPC) has a shallower HOMO at (– 5.5 eV) than CBP, and the TA spectrum of 5wt% P_3TTM:TAPC only shows an anion PIA response as shown in Supplementary Fig. 11, since TAPC-P_3TTM CT excitons also form quickly, but the strong electron-donating ability of TAPC terminates the second CT process between the ground state radical and positively charged TAPC. As shown in the time resolved PL of TAPC film, molecular doublet emission is completely quenched, and it only shows host-dopant exciplex emission with quicker kinetics that does not have a MFE. Following this, in 0.1wt% doped polymethyl methacrylate (PMMA) films, only molecular doublet emission and D_1 PIA state are observed, similar to the diluted solution behaviour, because the radicals are dispersed enough and insulating PMMA cannot act as a mediator. As a result, both direct and indirect CT channels are turned off.

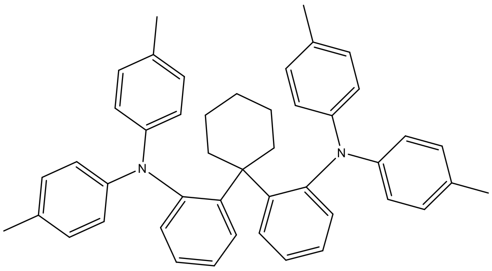

Molecular structure of TAPC

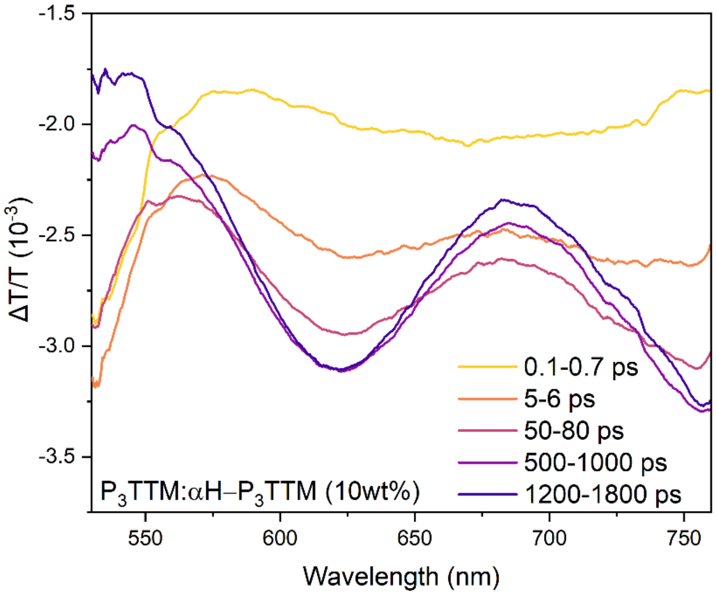

Supplementary Fig. 13. ps TA spectrum in the visible region of P_3TTM:αH-P_3TTM (10wt%) (λ_{ex}= 400 nm, 45 μJcm^{-2} per pulse). In addition to the PIA shoulder of molecular D_1 is probed, radical anions and cations PIAs are also probed.

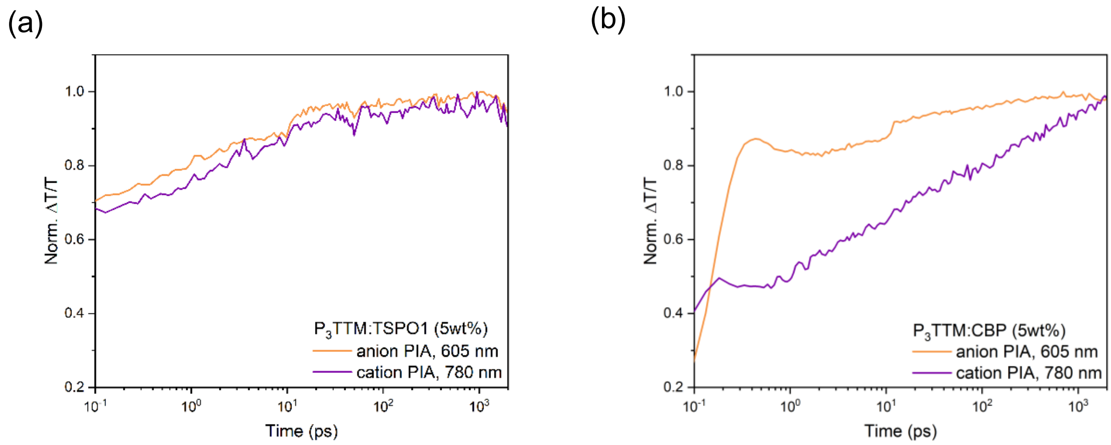

Supplementary Fig. 14 The normalised ps visible TA kinetics of anion (605 nm) and cation (780 nm) PIAs of **(a)** P_3TTM:TSPO1 (5wt%) and **(b)** P_3TTM:CBP (5wt%) reach the maximum within 2 ns. The anion and cation have the same generation kinetics in the TSPO1, while, in the CBP, the anion PIA is seen before the cation PIA due to the rapid CT between spinless CBP-P_3TTM.

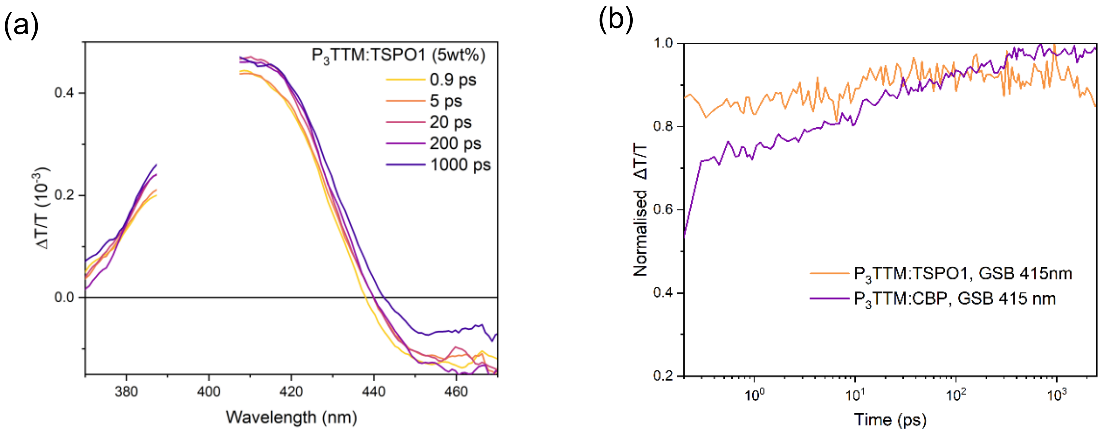

Supplementary Fig. 15 (a) ps TA spectrum in the ultra-violet region of P3TTM:TSPO1 (λ_{ex}= 400 nm, 13 μJcm^{-2} per pulse) shows a strong a ground state bleach (GSB) signal in the region of 360 nm to 430 nm and molecular PIA shoulder in the 430 nm to 480 nm. Similar to the CBP film, the GSB signal of P3TTM increases with time, while the broad shoulder PIA reduces. The spectrum break is due to the pump laser scattering. **(b)** The normalized ps TA kinetics of GSB at 415 nm of P3TTM in TSPO1 and CBP. The GSB signal of both films increases with time and reaches the maximum at *ca.* 1-2 ns.

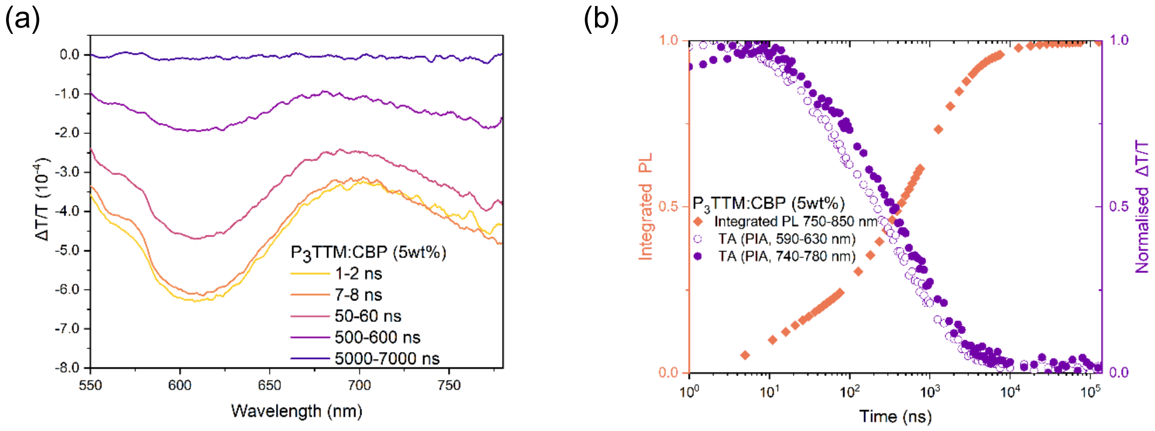

Supplementary Fig. 16 (a) ns TA spectrum of P3TTM in CBP in the visible region (λ_{ex}= 532 nm, 458 μJcm^{-2} per pulse). The spectrum has two PIA features in 590-630 nm and 740-780 nm regions for the anions and cations. **(b)** A comparison of PIA kinetics and red-shifted emission band kinetics of film to reveal the nature of PIA states. There is no excited state absorption when all photons are emitted, confirming the electron-hole recombination from anion and cation pairs are responsible for the red-shifted emission.

Estimation of inter-radical charge transfer quantum yield

Here, we estimate the charge transfer quantum yield using GSB signal in CBP film as an example. The number of photogenerated doublet exciton can be calculated by pump fluence and thin film absorption. At a pump fluence of 13 μJcm^{-2}, 3.82×10^{17} doublet excitons are generated upon photoexcitation, resulting in a GSB signal at 0.35 mΔOD. Within 2 ns, the GSB grows to 0.49 mΔOD, indicating that an additional 1.51×10^{17} P_3TTM molecules are bleached due to intermolecular charge transfer. Thus, there are 1.51×10^{17} cation generated, corresponding to 1.51×10^{17} radical pairs. The quantum yield of charge transfer then is calculated by:

$$\phi(CT) = \frac{no.of\ radical\ pairs}{no.of\ excitons\ on\ photoexcitation} = \frac{1.51 \times 10^{17}}{3.82 \times 10^{17}} = 40\%$$

The charge transfer quantum yield is relatively high, especially considering that the film is doped with only 5wt% P_3TTM. Furthermore, since approximately 20% of the excitons recombine and relax to the ground state within 2 ns, the charge transfer quantum yield may be even higher.

S6. Magneto-photoluminescence of P_3TTM

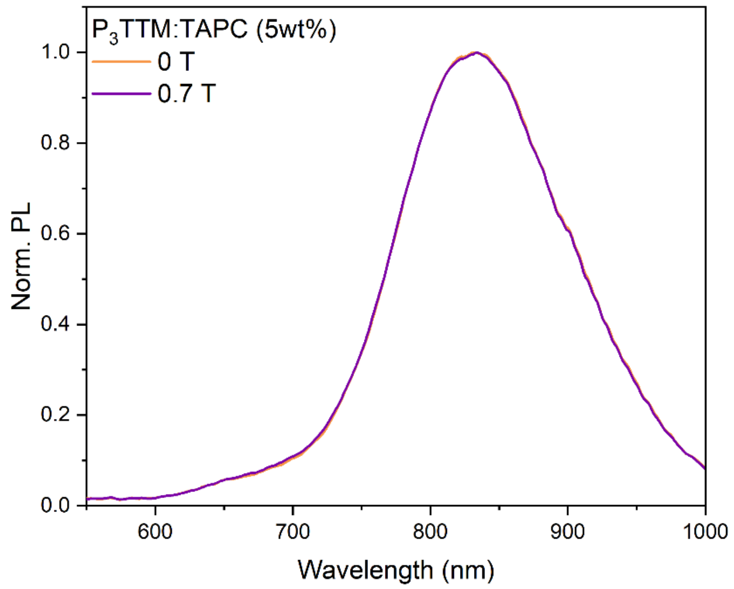

Supplementary Fig. 17 MagPL spectrum of P_3TTM:TAPC (5wt%) under 0 T and 0.7 T at room temperature, there is no significant change in PL spectra. Molecular emission is completely quenched, while only exciplex emission is seen.

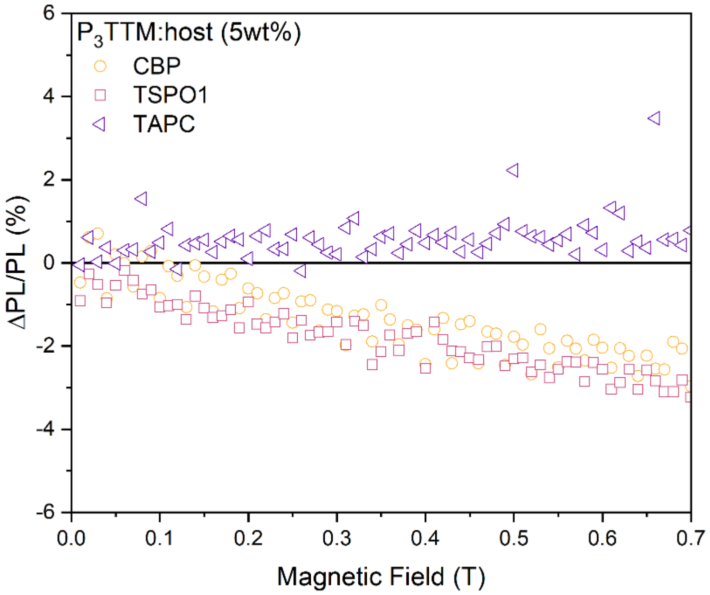

Supplementary Fig. 18 The magnetic field dependence of the ΔPL/PL (for the PL from 600 to 840 nm) which is calculated by $\frac{PL\ (0.7\ T)-PL(0\ T)}{PL\ (0\ T)}$. In CBP and TSPO1, ΔPL/PL reduces with the field linearly, since it modulates the spin state of molecule pairs which control the CT channel. In TAPC, the exciplex emission does not respond to the magnetic field.

S7. Quantum-chemical calculations

From the P3TTM's X-ray crystal structure, molecular pairs in close contact were selected according to geometrical criteria (e.g., showing centre of mass distances < 25 Å and atom-atom distances < 5 Å). The dimers were subjected to a broken-symmetry Density Functional Theory (DFT) optimization by using the B3LYP exchange-correlation functional and the 6-31G(d) basis set. Excited-state calculations based on TDA TDDFT were carried out on the optimized dimers with the LC-ωhPBE functional and the 6-311G(d,p) basis set, by applying the *screened* range-separated hybrid procedure and choosing a dielectric constant typical of toluene, ε = 2.37.

As previously done in other works, electronic couplings between the singlet localized P3TTM exciton (LE) and singlet charge-transfer (CT) state $V_{LE\text{-}CT}$, as well as those between the CT and ground state (GS) $V_{CT\text{-}GS}$, were computed within the generalized Mulliken-Hush (GMH) scheme.[3,4] In the two-states model, the equation reads as follows:

$$V_{i-f} = \frac{\Delta E_{if}\vec{\mu}_{if}}{\sqrt{\Delta\mu_{if}^2 + 4\vec{\mu}_{if}^2}}$$

where ΔE_{if} is the energy difference, $\vec{\mu}_{if}$ the transition dipole moment, and $\Delta\mu$ the dipole moments difference between state *i* and *f*.

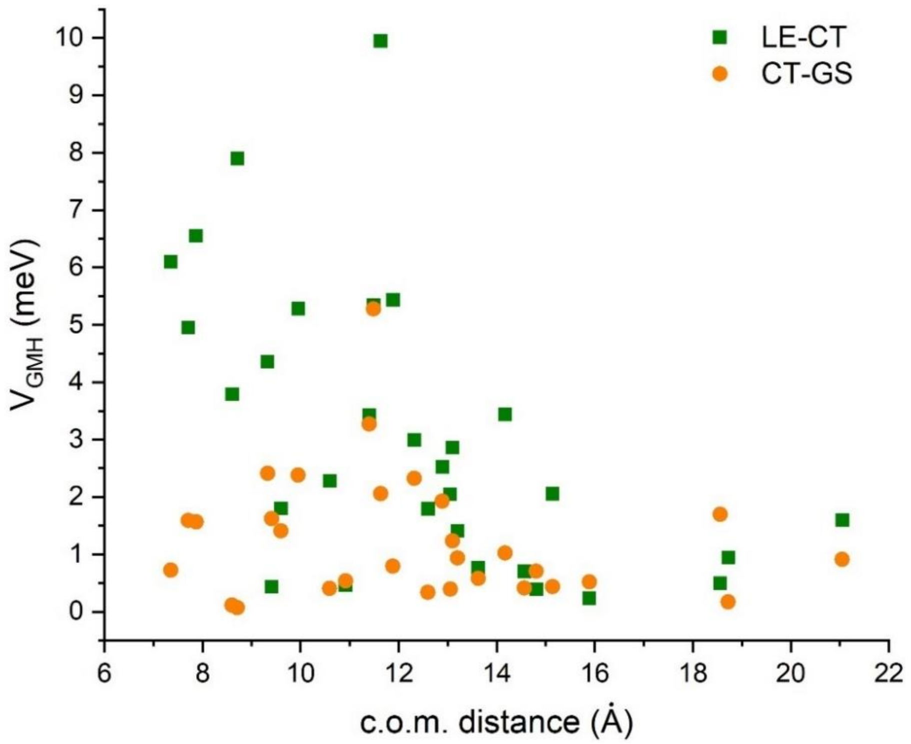

Supplementary Fig. 19 Distribution of LE-CT and CT-GS electronic couplings (in meV) as a function of the center of mass distance between molecular pairs.

Nonradiative (both charge dissociation and charge recombination) decay rates were estimated by using the phenomenological Marcus-Levich-Jortner theory:

$$\kappa_{nr} = \frac{2\pi}{\hbar} V_{i-f}^{2} \sqrt{\frac{1}{4\pi\lambda_s k_B T}} \times \sum_{n} \left\{ \exp(-S_{eff}) \frac{S_{eff}^{n}}{n!} \times exp \left[-\frac{\left(\Delta E_{if} + \lambda_s + n\hbar\omega_{eff}\right)^2}{4\lambda_s k_B T} \right] \right\}$$

where $\hbar$ is the Planck's constant, k_B the Boltzmann's constant, T the temperature set at 300 K, λ_s the outer-sphere reorganization energy set to 0.1 eV. In the model, S_{eff} and ω_{eff} are the effective Huang-Rhys factor and vibrational frequency, respectively. In this work, as effective vibrational frequency we set $\omega_{eff} = 1700\ cm^{-1}$, typical of a C=C double bond stretching. Our approximation is fully justified by computing explicitly the Huang-Rhys for each species (neutral, cation, anion, excited) of the isolated P_3TTM molecule (in the C_3 symmetry). As shown in the insets below, an intense activity of the high-frequency normal modes was observed between 1500 and 1700 cm^{-1}, which contribute most to either the transfer, adding, or removal of an electron.

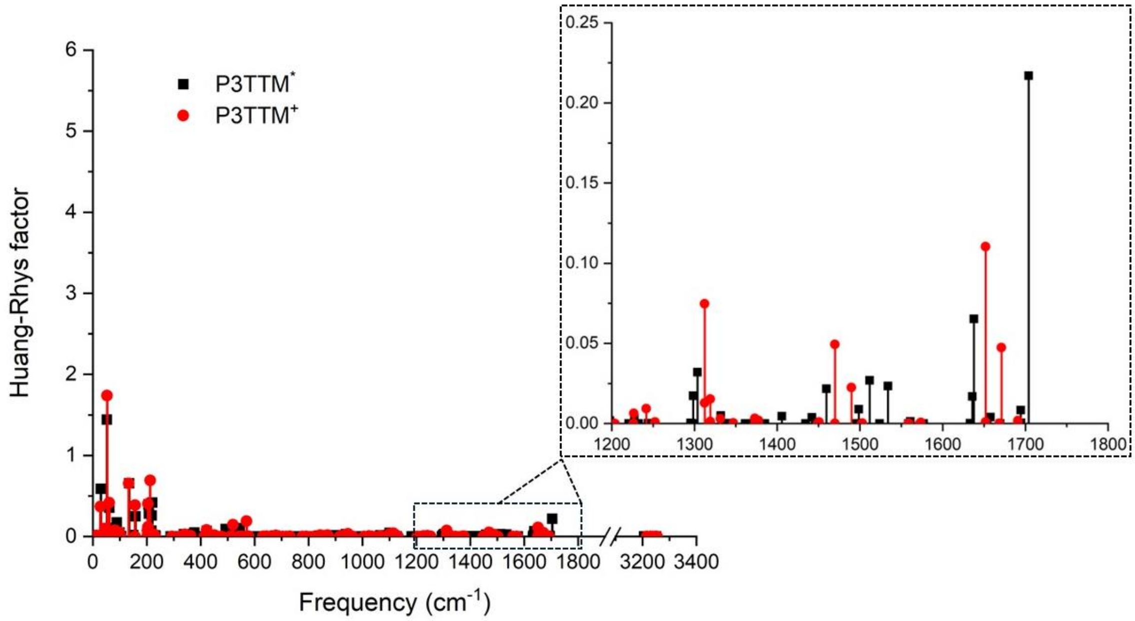

Supplementary Fig. 20 The Huang-Rhys factors of the full frequency spectrum of the excited P_3TTM^* and the cation P_3TTM^+ species. The inset reports a zoom of the high-frequency region.

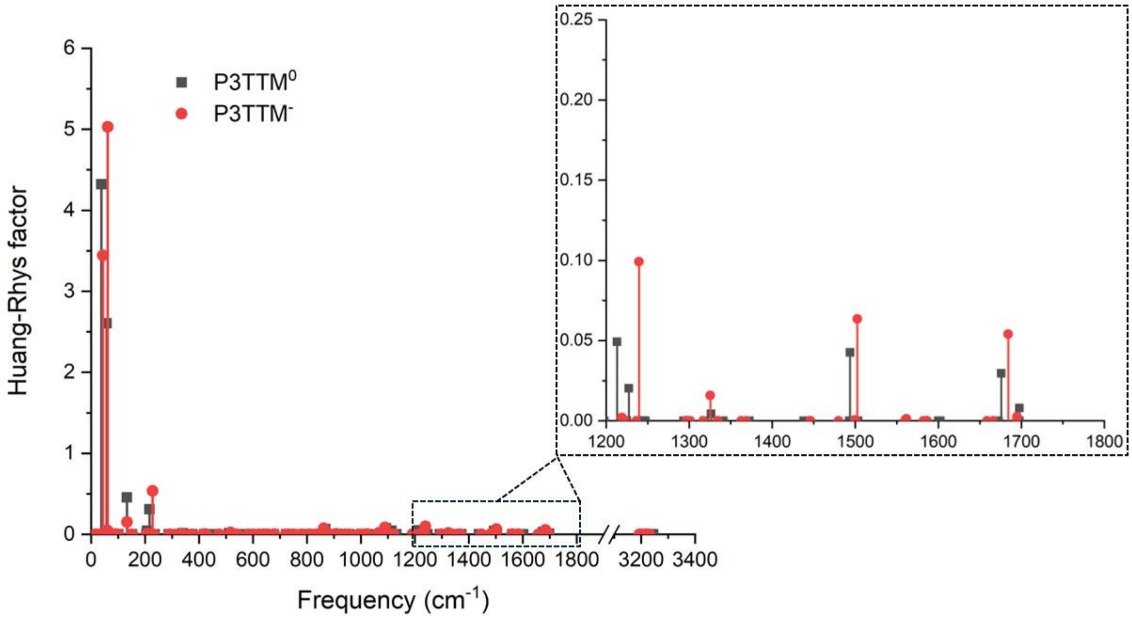

Supplementary Fig. 21 The Huang-Rhys factors of the full frequency spectrum of the neutral P_3TTM^0 and the anion P_3TTM^- species. The inset reports a zoom of the high-frequency region.

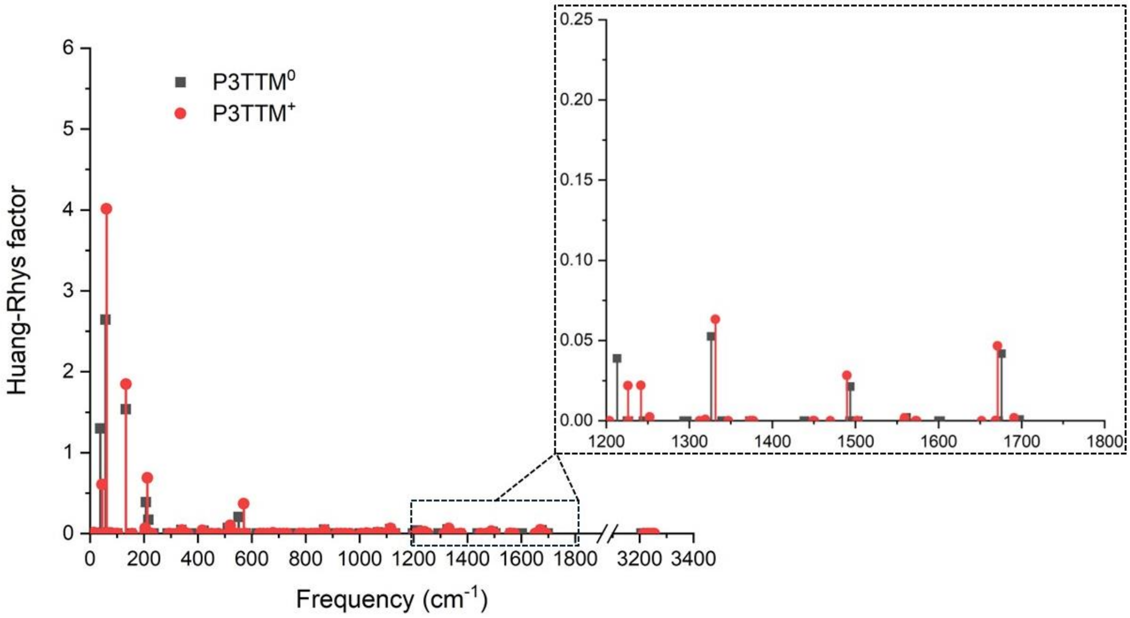

Supplementary Fig. 22 The Huang-Rhys factors of the full frequency spectrum of the neutral P_3TTM^0 and the cation P_3TTM^+ species. The inset reports a zoom of the high-frequency region.

For each normal mode, the Huang-Rhys factor is defined as:

$$S_i = {}^{\lambda_i}/_{\hbar\omega_i}$$

where λ_i represents the inner-sphere reorganization energy, computed quantum-mechanically for an isolated P_3TTM molecule (X) at the (TD) DFT ωB97X-D/6-31G(d,p) level of theory, depending on the process under investigation. For instance, for intermolecular charge dissociation, assuming that an electron has been transferred from the excited molecule, the corresponding process is: $X_1^* + X_2 \rightarrow X_1^+ + X_2^-$, while for charge recombination to the GS:

$X_1^+ + X_2^- \rightarrow X_1 + X_2$. As a result of the 4-points approach (see Table S1), the total internal reorganization energy upon LE → CT charge dissociation was estimated at 0.33 eV, while for the CT → GS charge recombination process λ_{tot} is 0.28 eV.

	λ_1 (eV)		λ_2 (eV)	λ_{tot} (eV)
$X_1^* \rightarrow X_1^+$	0.20	$X_2 \rightarrow X_2^-$	0.13	0.33
$X_1^+ \rightarrow X_1$	0.15			0.28

Supplementary Table 1 The reorganization energy of each process, where $\lambda_{tot} = \lambda_1 + \lambda_2$.

Radiative decay rates from the CT to GS were computed as:

$$\kappa_r = \frac{4}{3}\left(\frac{\omega_{if}^3 \vec{\mu}_{if}^2}{3\pi\varepsilon_0 \hbar c^3}\right)\left(\frac{n(n^2+2)^2}{9}\right)$$

where ω_{if} is the transition frequency between state i and f, ε_0 is the vacuum permittivity, c is the light speed. The last term between parenthesis is a correction for the local electric field effect, being $n = 1.497$ the refractive index of toluene at 20°C.

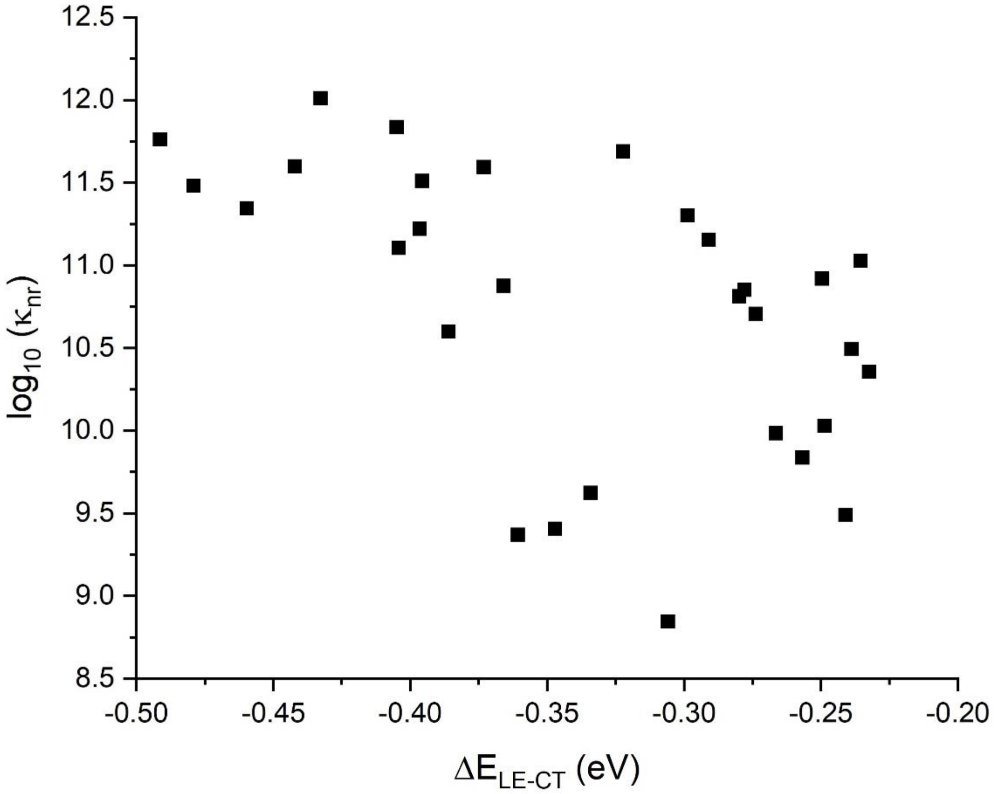

Supplementary Fig. 23 The nonradiative charge dissociation rates (reported on a logarithmic scale) as a function of the energy difference between the LE and CT state. Raw data are reported below in Table S2. The larger the driving force, the faster the charge dissociation, as predicted within the Marcus-Levich-Jortner framework.

ΔE_{LE-CT} (eV)	μ_{CT} (D)	μ_{LE} (D)	$\vec{\mu}_{LE-CT}$ (D)	V_{LE-CT} (meV)	κ_{nr}^{LE-CT} (s^{-1})	τ_{nr}^{LE-CT} (ps)
-0.49	41.9	5.9	0.6	6.6	5.8E+11	2
-0.24	66.1	3.5	0.8	3.0	1.1E+11	9
-0.46	48.3	6.1	0.5	4.4	2.2E+11	5
-0.40	48.1	5.7	0.7	5.3	3.2E+11	3
-0.28	67.4	9.5	0.6	2.1	7.1E+10	14
-0.33	54.8	6.9	0.1	0.5	4.2E+09	237
-0.23	65.8	9.0	0.4	1.4	2.3E+10	44
-0.36	60.0	9.4	0.1	0.4	2.3E+09	426
-0.44	46.7	4.8	0.7	6.1	4.0E+11	3
-0.43	46.3	5.5	1.2	9.9	1.0E+12	1
-0.48	41.8	6.2	0.5	4.9	3.0E+11	3
-0.37	53.8	5.2	0.4	2.3	7.5E+10	13
-0.39	51.2	8.6	0.3	1.8	4.0E+10	25
-0.24	65.8	8.8	0.5	1.6	3.1E+10	32
-0.27	67.4	9.4	0.5	1.8	5.1E+10	20
-0.40	46.1	9.7	0.3	3.4	1.3E+11	8
-0.26	67.8	8.9	0.2	0.7	6.9E+09	146
-0.41	47.7	3.6	1.0	7.9	6.8E+11	1
-0.30	64.1	8.1	0.8	3.4	2.0E+11	5
-0.31	69.1	9.5	0.1	0.2	7.0E+08	1426
-0.25	74.9	8.1	0.3	0.9	1.1E+10	94
-0.24	68.2	8.2	0.2	0.5	3.1E+09	323
-0.37	49.2	8.4	0.8	5.4	3.9E+11	3
-0.28	67.3	9.1	0.6	2.0	6.5E+10	15
-0.35	66.8	8.3	0.1	0.4	2.5E+09	393
-0.40	47.7	5.5	0.5	3.8	1.7E+11	6
-0.27	67.9	9.0	0.2	0.8	9.6E+09	104
-0.29	66.7	8.6	0.7	2.9	1.4E+11	7
-0.25	63.4	7.8	0.7	2.5	8.3E+10	12

Supplementary Table 2 Raw data regarding the charge dissociation process: the energy difference between the LE and CT states (being the latter lower in energy); the CT and LE dipole moments and the associated transition dipole moment (in D); the electronic coupling (in meV) computed within the GMH scheme; the nonradiative charge dissociation rate (in s^{-1}) and the corresponding lifetime (in ps). Out of 32 initially selected dimers, 3 were discarded as outliers.

As a reference for μ_{LE}, the computed lowest-energy (at 2.70 eV) excited state dipole moment of the monomer doublet P_3TTM with C_3 symmetry (unrestricted DFT LC-ωhPBE/6-311G(d,p) in toluene level of theory) is 5.8 D.

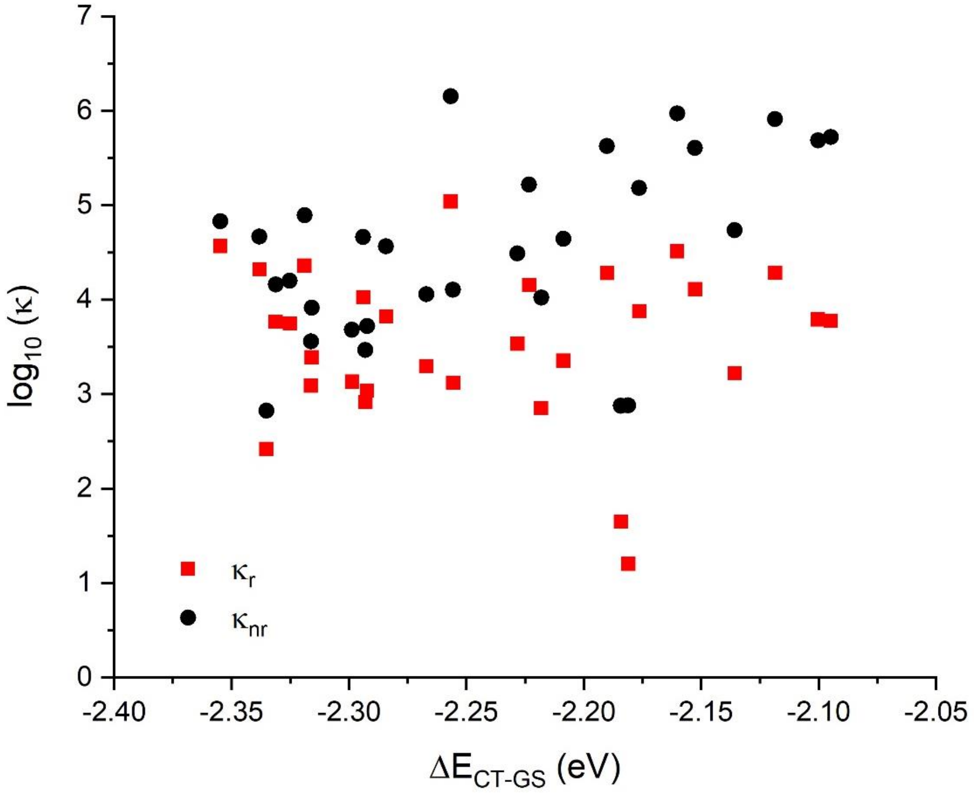

Supplementary Fig. 24 The radiative and nonradiative charge recombination rates (reported on a logarithmic scale) as a function of the energy difference between the CT and GS. Raw data are reported below in Table S3. The smaller the CT-GS energy difference, the faster the nonradiative recombination rates (over the radiative ones), as a result of the energy-gap law.

ΔE_{CT-GS} (eV)	μ_{GS} (D)	μ_{CT} (D)	$\vec{\mu}_{CT-GS}$ (mD)	V_{CT-GS} (meV)	κ_{r}^{GS-CT} (s^{-1})	κ_{nr}^{GS-CT} (s^{-1})	τ_{nr}^{GS-CT} (μs)
-2.09	0.3	41.9	31	1.6	5.9E+03	5.3E+05	2
-2.35	0.3	66.1	65	2.3	3.7E+04	6.7E+04	15
-2.12	0.5	48.3	56	2.4	1.9E+04	8.2E+05	1
-2.19	0.5	48.1	52	2.4	1.9E+04	4.2E+05	2
-2.30	0.6	67.4	13	0.4	1.4E+03	4.8E+03	209
-2.26	0.4	54.8	13	0.5	1.3E+03	1.3E+04	78
-2.33	0.3	65.8	26	0.9	5.9E+03	1.4E+04	69
-2.22	0.7	60.0	44	1.6	1.4E+04	1.6E+05	6
-2.14	0.5	46.7	16	0.7	1.7E+03	5.4E+04	18
-2.15	0.3	46.3	44	2.1	1.3E+04	4.0E+05	2
-2.10	0.3	41.8	32	1.6	6.2E+03	4.8E+05	2
-2.22	0.3	53.8	10	0.4	7.1E+02	1.1E+04	95
-2.18	0.3	51.2	33	1.4	7.6E+03	1.5E+05	7
-2.33	0.3	65.8	26	0.9	5.6E+03	1.6E+04	63
-2.29	0.6	67.4	10	0.3	8.2E+02	2.9E+03	342
-2.16	0.4	46.1	70	3.3	3.3E+04	9.3E+05	1
-2.32	0.5	67.8	12	0.4	1.2E+03	3.6E+03	276
-2.18	0.2	47.7	1	0.1	1.6E+01	7.6E+02	1318
-2.28	0.4	64.1	29	1.0	6.6E+03	3.7E+04	27
-2.27	0.9	69.1	16	0.5	2.0E+03	1.1E+04	88
-2.34	0.7	74.9	5	0.2	2.6E+02	6.7E+02	1494
-2.34	0.5	68.2	50	1.7	2.1E+04	4.6E+04	22
-2.21	0.4	49.2	18	0.8	2.3E+03	4.4E+04	23
-2.29	0.6	67.3	12	0.4	1.1E+03	5.3E+03	190
-2.23	0.9	66.8	22	0.7	3.4E+03	3.1E+04	33
-2.18	0.2	47.7	3	0.1	4.5E+01	7.5E+02	1336
-2.32	0.4	67.9	17	0.6	2.5E+03	8.2E+03	122
-2.29	0.6	66.7	36	1.2	1.1E+04	4.6E+04	22
-2.32	0.3	63.4	53	1.9	2.3E+04	7.8E+04	13

Supplementary Table 3 Raw data regarding the charge recombination process: the energy difference between the CT and GS; the GS and CT dipole moments (in D) and the associated transition dipole moment (in mD); the electronic coupling (in meV) computed within the GMH scheme; the radiative and nonradiative charge recombination rate (in s^{-1}), for the latter the corresponding lifetime (in μs). Out of 32 initially selected dimers, 3 were discarded as outliers.

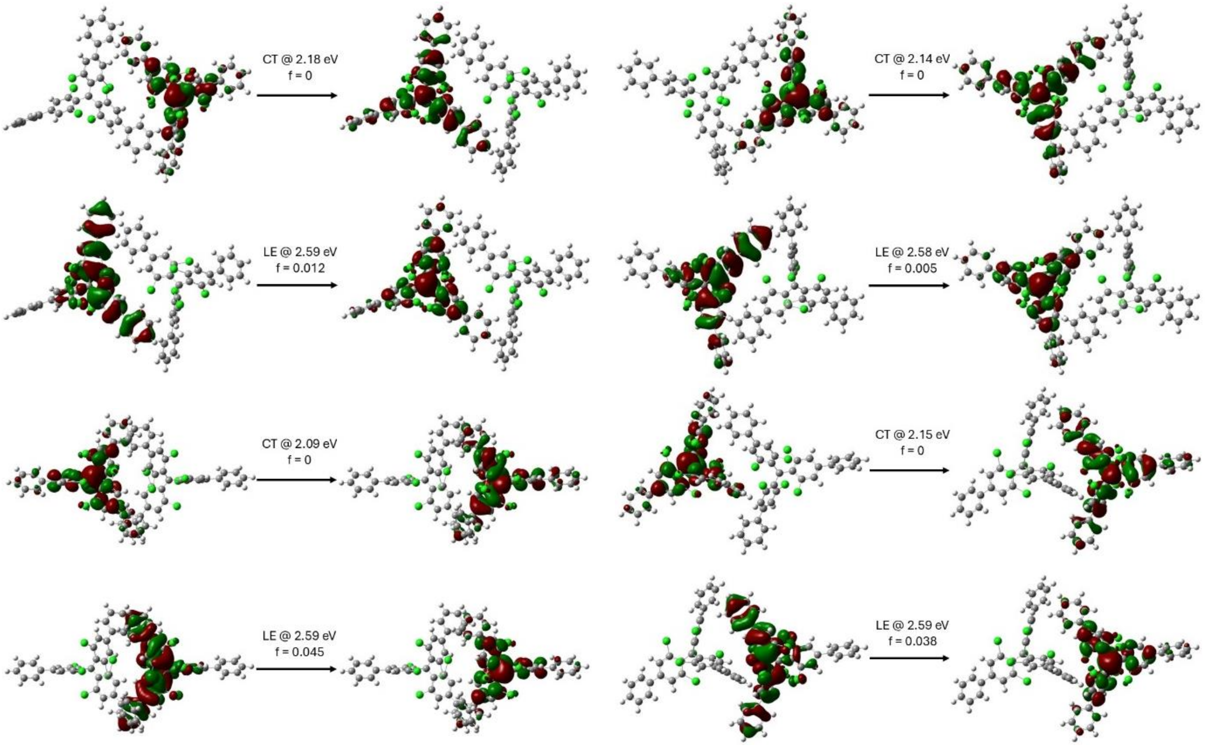

Supplementary Fig. 25 Four representative dimers and their singlet CT *vs* LE hole-particle natural transition orbitals. Energies of the excited states and their oscillator strengths (*f*) are also reported.

S8. X-Ray crystallography

X-Ray crystallography and X-ray crystal structures for P_3TTM (CCDC 2305974), 2,6-X_3TTM (CCDC 2305967) and 3,5-X_3TTM (CCDC 2305971) have been reported previously. [2] These data can be obtained free of charge via www.ccdc.cam.ac.uk/data_request/cif, or by emailing data_request@ccdc.cam.ac.uk, or by contacting The Cambridge Crystallographic Data Centre, 12 Union Road, Cambridge CB2 1EZ, United Kingdom; fax: +44 1223 336033. We visualize the crystal packing and the closest contacts between terminal phenyl groups in these radicals in the figures below.

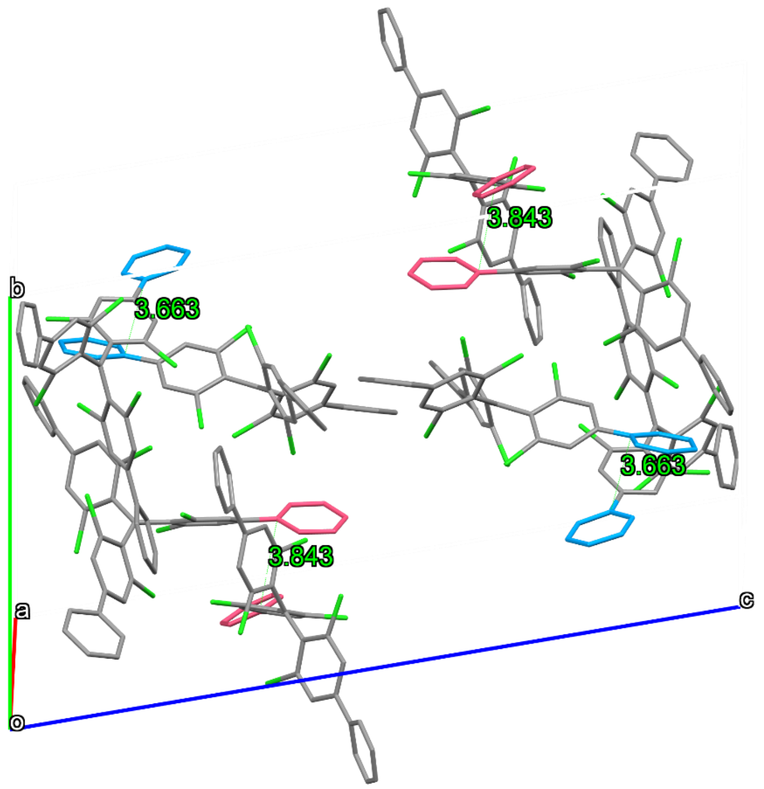

Supplementary Fig. 26 A unit cell of X-ray crystal structure of P_3TTM viewed along crystallographic a* axis. The two closest contacts are calculated between carbon 1-positions of the terminal phenyl rings (the corresponding rings are coloured blue and red). Hydrogens are omitted for clarity.

S9. The concentration effect on the intermolecular charge transfer

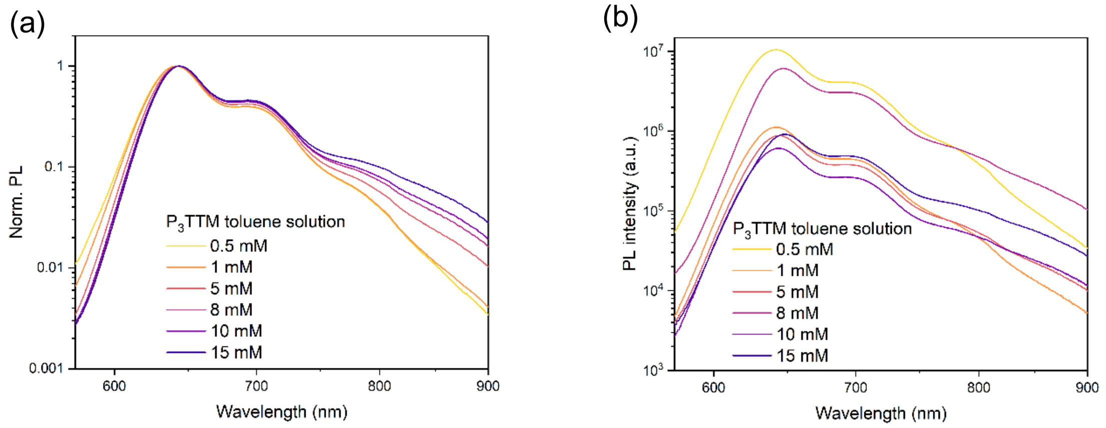

Supplementary Fig. 27 (a) The normalised (relative to 645 nm PL) PL spectra and **(b)** PL spectra (λ_{ex}=400 nm) in log scale of P3TTM toluene solution with different concentrations. The red-shifted emission increases relative to monomer emission from 0.5mM to 15mM, while overall emission is quenched with increasing concentrations.

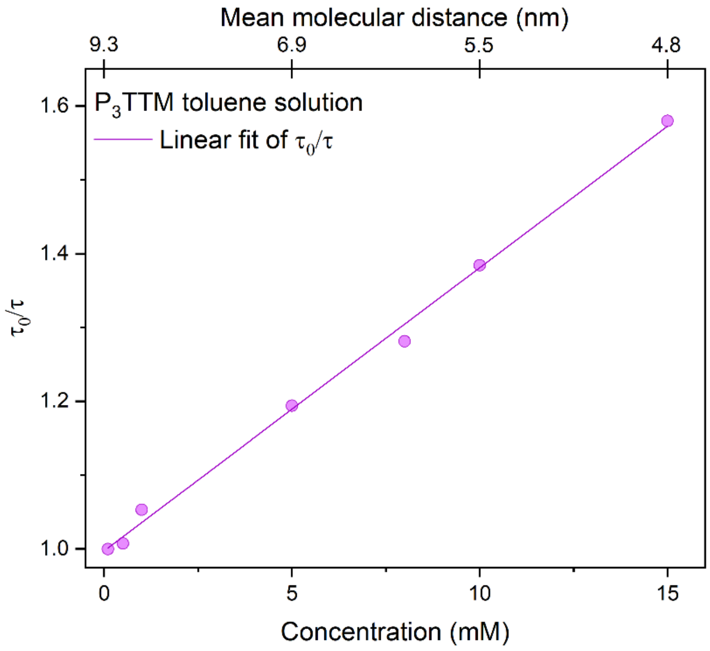

Supplementary Fig. 28 The Stern-Volmer plot for P3TTM monomer PL quenching shows a linear relationship with the solution concentration, indicating a dynamic quenching (with a bimolecular quenching constant k_q of 4.12×10^9 $M^{-1}s^{-1}$). The τ_0 is lifetime of P3TTM in 0.1 mM toluene solution measured by the time-correlated single photon counting (TCSPC), τ is lifetime of P3TTM in more concentrated solution.

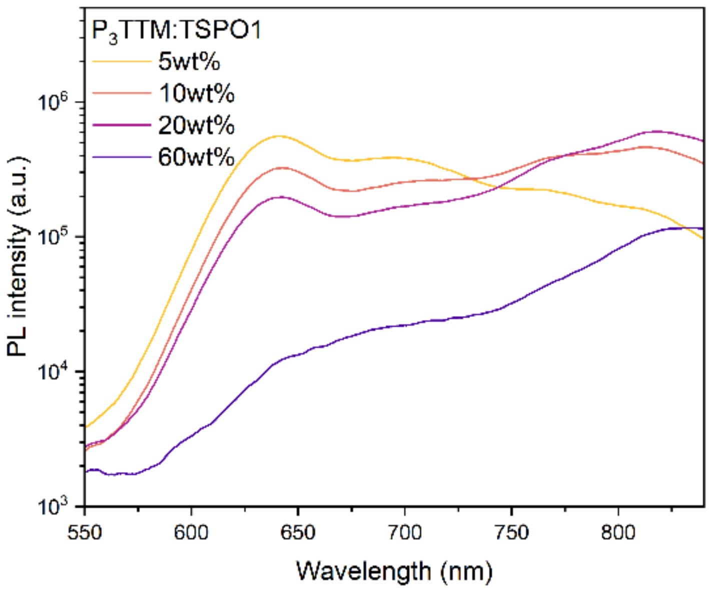

Supplementary Fig. 29 The PL intensity spectra in log scale of P_3TTM:TSPO1 with different doping concentrations (λ_{ex}= 400 nm). The red-shifted emission increases relative to monomer emission from 5wt% to 60 wt% corresponding to a mean inter-molecular distance from 2.8 nm to 1.2 nm. (assume film density is 1g/cm^3).[5] A reduction of film transmittance from 80% to 20% should be considered for increasing doping concentration so more photons are absorbed for heavily doped films (see Supplementary Table 4 below).

doping concentration	5 wt%	10 wt%	20 wt%	60 wt%
**PLQE (600-850nm)**	4.73%	1.33%	0.28%	0.06%
**PLQE (600-750nm)**	3.38%	0.66%	0.07%	0.01%
**PLQE (750-850nm)**	1.35%	0.67%	0.21%	0.05%

Supplementary Table 4 The PLQE of P_3TTM:TSPO1 with different doping concentrations (λ_{ex}= 400 nm) and different PL regions of doublet excitons and CT state.

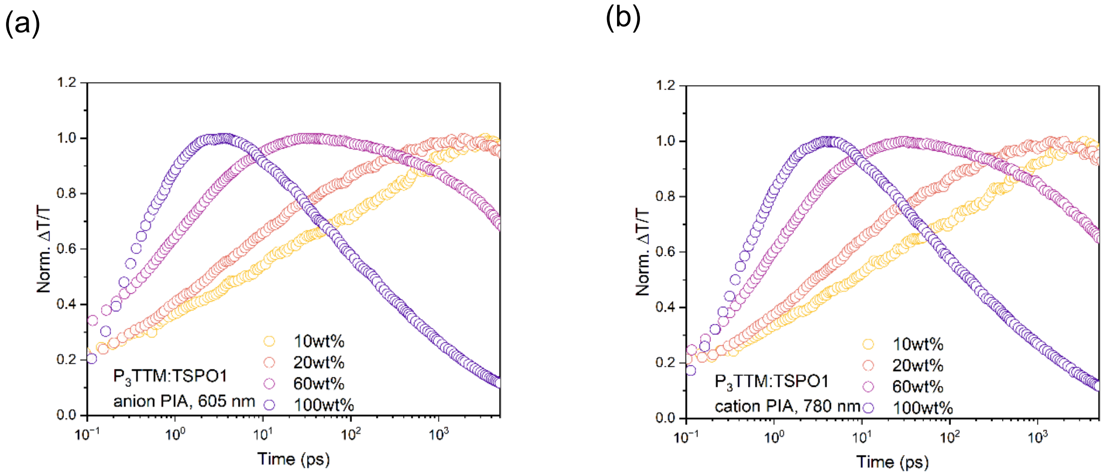

Supplementary Fig. 30 The normalised ps TA kinetics (λ_{ex}= 400 nm, 9 μJcm^{-2} per pulse) of **(a)** anion PIA (at 605 nm) and **(b)** cation PIA (at 780 nm) signals of P_3TTM:TSPO1 with different doping concentrations from 10wt% to 100wt% for a CT distance range from 2.2 nm to 1 nm. The rate of radical ion generation increases with the film doping concentrations (i.e. CT distance).

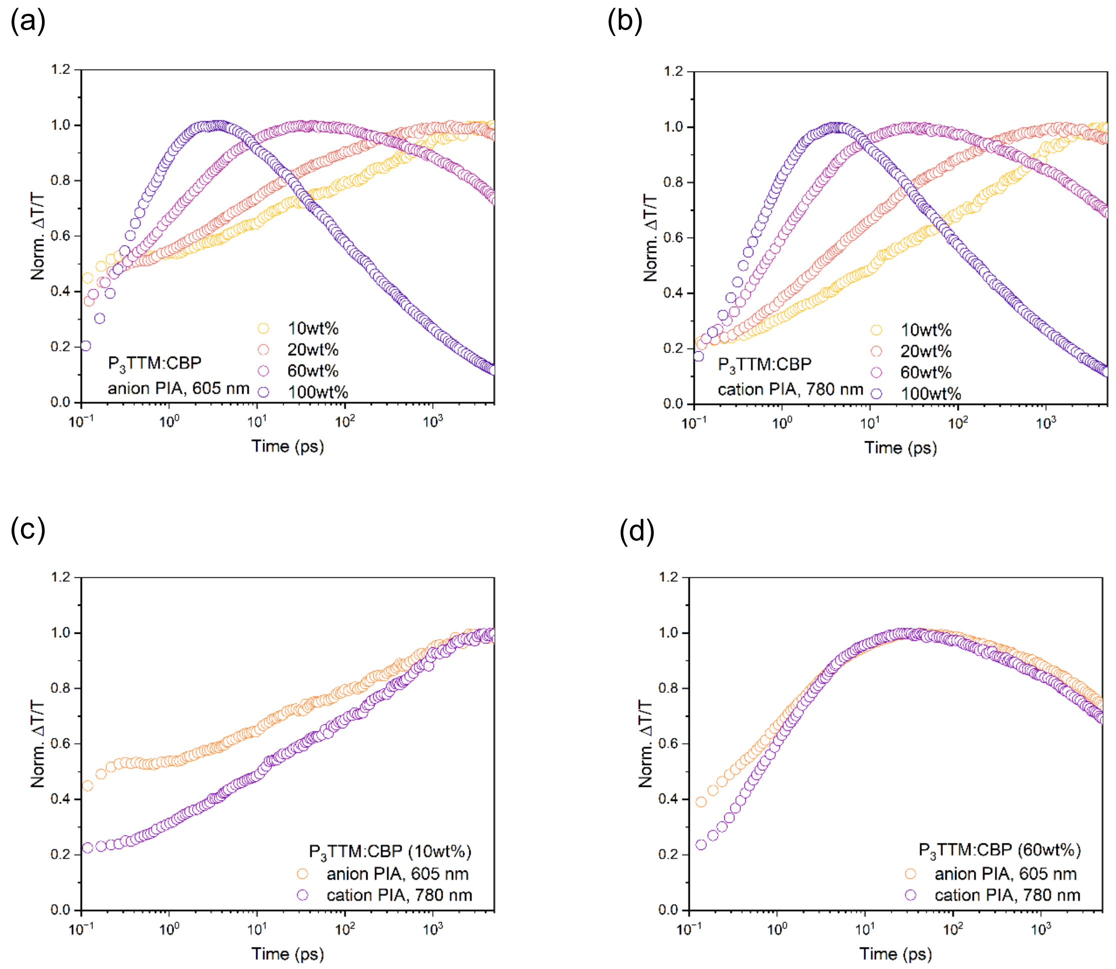

Supplementary Fig. 31 The normalised ps TA kinetics (λ_{ex}= 400 nm, 9 μJcm^{-2} per pulse) of **(a)** anion PIA (at 605 nm) and **(b)** cation PIA (at 780 nm) signals of P_3TTM:CBP with different doping concentrations from 10wt% to 100wt% for a CT distance range from 2.2 nm to 1 nm. The rate of radical ion generation increases with the film doping concentrations (i.e. CT distance). A comparison of normalised anion and cation PIA kinetics of **(c)** 10wt% and **(d)** 60wt% doped films. Two PIA kinetics become closer because the possibility of direct charge transfer between P_3TTM increases with increasing doping concentration.

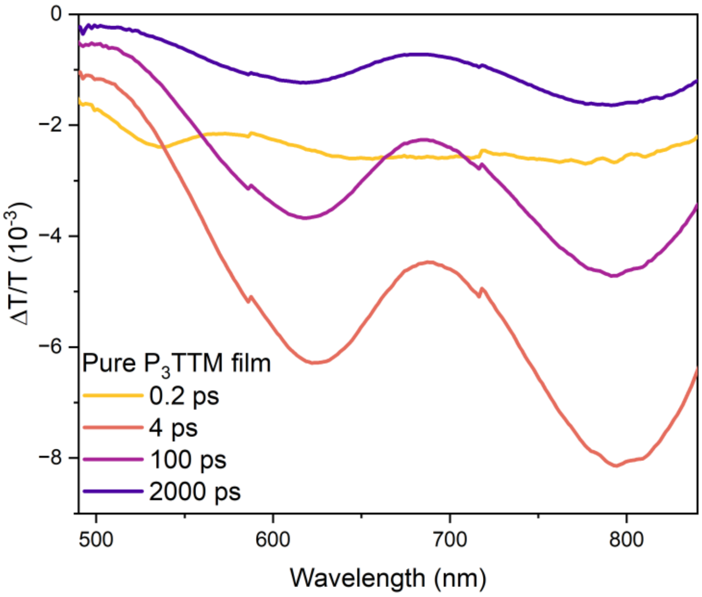

Supplementary Fig. 32 The visible ps TA spectrum of P3TTM without host matrix (λ_{ex}= 400 nm, 13 μ$J cm^{-2}$ per pulse) shows a similar intermolecular CT in between radicals, which forms anions (PIA, 575 nm to 625 nm) and cations (PIA, beyond 750 nm to 840 nm).

S10. Device characterisation

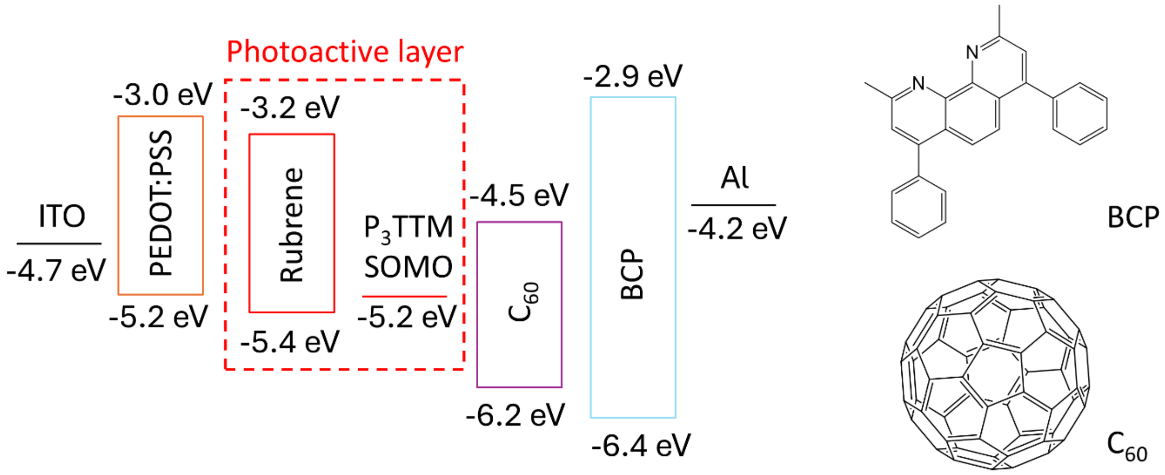

Supplementary Fig. 33 Energy levels for devices with a structure of ITO(150 nm)/PEDOT:PSS (40 nm)/photoactive layer (80 nm)/C_{60}(20 nm)/BCP (5 nm)/Al (100 nm) for P_3TTM and rubrene devices (BCP: bathocuproine, C_{60}: fullerene).

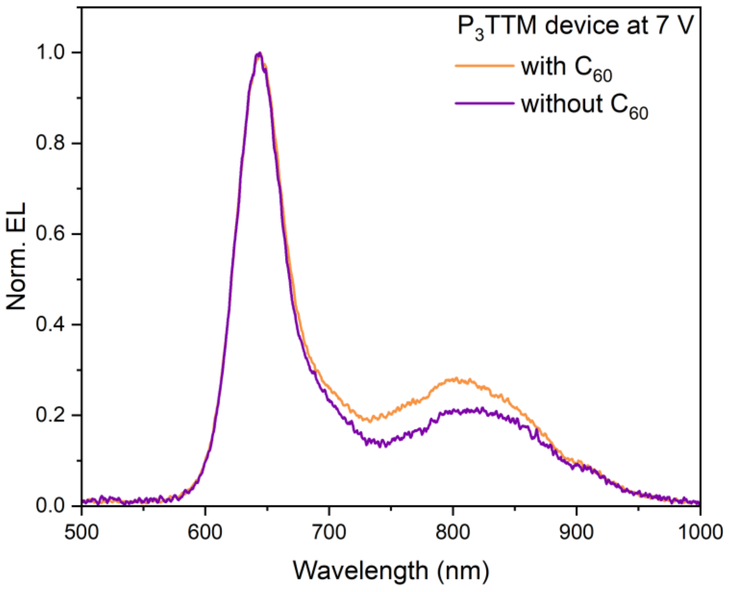

Supplementary Fig. 34 The electroluminescence spectrum of P_3TTM devices with or without C_{60} layer at 7V. It shows both molecular exciton emission and charge transfer state emission under electrical injection.

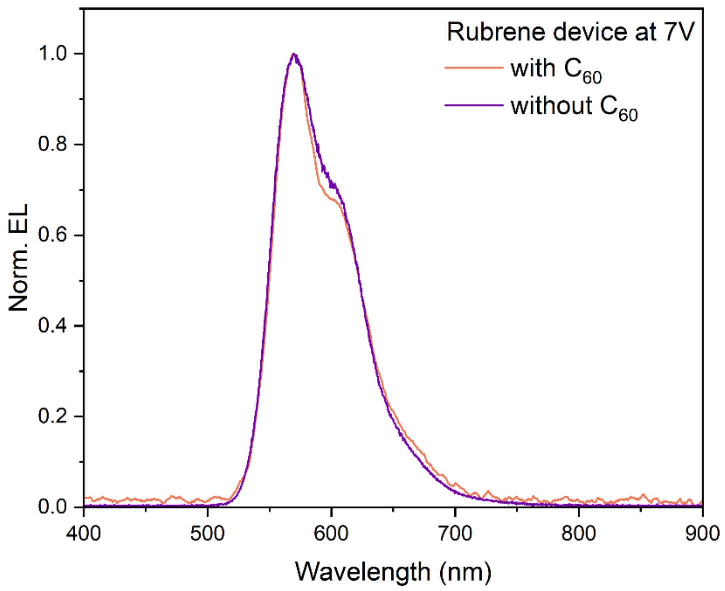

Supplementary Fig. 35 The electroluminescence spectrum of rubrene devices with or without C_{60} layer at 7V. It shows only molecular exciton emission at 570 m under electrical injection.

.

S11. Reference

1 Murto, P. *et al.* Mesitylated trityl radicals, a platform for doublet emission: symmetry breaking, charge-transfer states and conjugated polymers. *Nat. Commun.* **14**, 4147 (2023).

2 Murto, P. *et al.* Steric Control of Luminescence in Phenyl-Substituted Trityl Radicals. *J. Am. Chem. Soc.* **146**, 13133-13141 (2024).

3 Gillett, A.J., Privitera, A., Dilmurat, R. *et al.* The role of charge recombination to triplet excitons in organic solar cells. *Nature* **597**, 666–671 (2021).

4 Londi, G. et al. Fate of low-lying charge-transfer excited states in a donor:acceptor blend with a large energy offset. *J. Phys. Chem. Lett.* **11**, 10219-1026 (2020).

5 Xiang, H. *et al.* Method for measurement of the density of thin films of small organic molecules. *Rev. Sci. Instrum.* **78**, 034104 (2007).
